# Efficacy and safety of tirzepatide in patients with type 2 diabetes: A systematic review and meta-analysis

**DOI:** 10.3389/fphar.2022.1016639

**Published:** 2022-10-28

**Authors:** Yan Tang, Lin Zhang, Yuping Zeng, Xia Wang, Mei Zhang

**Affiliations:** ^1^ Department of Laboratory Medicine, West China Hospital, Sichuan University, Chengdu, China; ^2^ General Practice Ward/International Medical Center Ward, General Practice Medical Center, West China Hospital, Sichuan University, Chengdu, China

**Keywords:** type 2 diabetes, tirzepatide, HbA1c, weight loss, hypoglycemia, meta-analysis

## Abstract

**Purpose:** A systematic review and meta-analysis was conducted to combine the data available from clinical trials and evaluate the clinical efficacy and safety of tirzepatide in people with type 2 diabetes (T2D).

**Methods:** We systematically searched the MEDLINE, Embase, Cochrane Library, and clinical trials registries (https://clinicaltrials.gov) up to 25 March 2022 for randomized controlled trials (RCTs) that compared tirzepatide with placebo or active hypoglycemic drugs in subjects with T2D. Heterogeneity was judged by the *I* ^2^ value and Cochran’s Q test. The randomized effects model was adopted to calculate risk ratios and weighted mean differences (WMDs). The primary outcome was the change from baseline in HbA1c levels. Secondary efficacy endpoints were fasting serum glucose (FSG), change of body weight, blood pressure, fasting lipid profiles, and safety indexes.

**Results:** Six trials comprising 6,579 subjects (4,410 in the tirzepatide group and 2,054 in the control group) fulfilled the pre-specified criteria and were included in the study. Tirzepatide treatment resulted in reducing HbA1c (WMD: -1.07%; 95% confidence intervals [CIs]: −1.44, −0.56), FSG (WMD, −21.50 mg/dl; 95% CI: −34.44, −8.56), body weight (WMD: −7.99 kg; 95% CI −11.36, −4.62), and blood pressure and ameliorated fasting lipid profiles, without increasing hypoglycemia, either as monotherapy or an add-on therapy. Tirzepatide increased the risk of gastrointestinal adverse events mainly in add-on therapy but not in terms of pancreatitis or cholelithiasis. Furthermore, tirzepatide presented a dose–response effect on the reduction in HbA1c and body weight and increase in nausea and vomiting.

**Conclusion:** In patients with type 2 diabetes, tirzepatide shows superior blood glucose control and weight loss performance, without an increased risk of hypoglycemia.

**Systematic Review Registration:** (https://www.crd.york.ac.uk/PROSPERO), identifier (CRD42022319442).

## 1 Introduction

Diabetes has become one of the most prevalent non-communicable chronic diseases, resulting in disabilities, expensive complications, and even shortening of life expectancy (Saeedi et al., 2019). Unfortunately, in the world, the prevalence rate of diabetes in the population of age 20–79 years was 10.5% in 2021, which was about 536 million 600 thousand; by 2045, it is expected to increase up to 12.2%, which will be about 783 million 200 thousand (Sun H. et al., 2022).

According to the pathophysiology of diabetes, different antihyperglycemic drugs have been developed for clinical application. Since human insulin was approved in 1982, the FDA has approved 59 drugs for controlling hyperglycemia (Dahlen et al., 2021). Among these antihyperglycemic agents, incretin-dependent T2D therapies play an important role. As a 30-amino-acid-peptide, glucagon-like peptide-1 (GLP-1) exerts biological effects as an incretin-stimulating hormone. The first batch of incretin-based T2D therapies was approved in 2005 and 2006. Since then, they have become more and more popular. GLP-1 receptor agonists (GLP-1 RAs) such as liraglutide, dulaglutide, and semaglutide are the forms of incretin-based T2D therapy, which are used increasingly and show excellent clinical benefits including decreased glucose and body weight, lowered cardiovascular risk, and reduced risk of hypoglycemia (Lyseng-Williamson, 2019). Glucose-dependent insulinotropic polypeptide (GIP), the other incretin hormone, is similar to GLP-1 and its receptor; however, GIP does not inhibit appetite and food intake (Holst and Rosenkilde, 2020). Thus, it is assumed that the combination of GLP-1 RA with other drugs acting on GIP receptors may produce more effective blood glucose control and weight loss.

Tirzepatide, a dual GIP and GLP-1 RA, is a polypeptide containing 39 amino acids, which is combined using a bioactive N-terminal GIP sequence and exenatide-like C-terminal sequence, and conjugated with a fatty acid chain similar to the semaglutide side chain to promote its binding with albumin, so as to prolong the half-life of the drug (Coskun et al., 2018). A series of clinical trials on tirzepatide have explored its efficacy and safety in the treatment of T2D and shown great potential in decreasing hemoglobin A1c (HbA1c) and body weight (Min and Bain, 2021).

There are two systematic reviews and meta-analyses on the evaluation of the effectiveness of tirzepatide on the treatment of diabetes (Bhagavathula et al., 2021; Dutta et al., 2021). They both included one randomized, double-blind, placebo-controlled clinical trial which appraised the efficacy and tolerability of tirzepatide in patients with type 2 diabetes lasting for 12 weeks (Frias et al., 2020). However, the dose regimens in the study were dose-escalation. The duration of each dose was 2–4 weeks, and the treatment time of tirzepatide at the target stable dose was only 4 weeks, which was too short to judge the efficacy of a specific dose. On the other hand, both the systematic reviews did not perform dose–response analysis. Finally, the results of SURPASS-5 were published in 2022 and were not included.

We performed and updated a systematic review and meta-analysis to evaluate the safety and efficacy of tirzepatide in patients with T2D.

## 2 Methods

### 2.1 Protocol

Our systematic review and meta-analysis was executed and reported in accordance with the Preferred Reporting Items for Systematic Reviews and Meta-Analyses (PRISMA) statement (Page et al., 2021). The protocol was registered in PROSPERO (No. CRD42022319442).

### 2.2 Search strategy

The Embase, MEDLINE, and Cochrane databases and clinical trials registries (https://clinicaltrials.gov) were comprehensively searched prior to 25 March 2022, without limitations on language, race, or country. The following terms were used: (Tirzepatide OR LY3298176 OR twincretin OR dual glucose-dependent insulinotropic polypeptide and glucagon-like peptide-1 receptor agonist) AND (type 2 diabetes OR diabetes) AND (randomized controlled trial). The search strategy was adjusted to meet the requirements of each database.

### 2.3 Inclusion and exclusion criteria

We included randomized controlled trials (RCTs), which lasted at least 12 weeks, that assessed and compared the efficacy and safety of tirzepatide with other hypoglycemic agents or placebos in T2D patients (≥18 years old), diagnosed according to the World Health Organization (1999) or American Diabetes Association (1997) criteria. Reviews, letters, case reports, nonhuman studies, editorials, commentaries, expert opinions, non-RCTs, and meta-analyses were excluded.

### 2.4 Outcome measures of efficacy and safety

The change in HbA1c from the baseline was considered the primary outcome of efficacy. The secondary endpoints included proportions of patients with HbA1c<7.0%, ≤6.5%, or <5.7%, blood glucose including fasting serum glucose (FSG), body weight profile (body weight change from baseline, participants with ≥5%, ≥10%, or ≥15% weight loss), fasting lipid profile (total cholesterol, triglycerides, and HDL cholesterol), homeostatic model assessment 2-insulin resistance (HOMA2-IR), blood pressure (systolic blood pressure and diastolic blood pressure), and adverse events including adverse events leading to treatment discontinuation, hypoglycemic events (blood glucose <70, 54 mg/dl or severe hypoglycemia), gastrointestinal events (nausea, diarrhea, dyspepsia, decreased appetite, vomiting, and constipation), pancreatitis, cholelithiasis, and major adverse cardiovascular event-4 (MACE-4) (a composite of cardiovascular death, myocardial infarction, stroke, and hospitalization for unstable angina). Severe hypoglycemia was defined as the onset of severe cognitive impairment that required the assistance of another person to actively take carbohydrates, glucagon, or other resuscitation measures.

### 2.5 Data extraction

Data extraction was carried out by two reviewers (XW and YZ) independently according to the inclusion and exclusion criteria. Any differences in the extracted data between the two reviewers were discussed and resolved by consensus. The following information was extracted: study characteristics, subjects’ baseline data on biological characteristics, interventions, efficacy, and safety results.

### 2.6 Risk of bias assessment

The Cochrane Collaboration’s Risk of Bias Tool with Review Manager (Higgins et al., 2011) was used to evaluate the methodological quality of the included RCTs, which included random sequence generation (selection bias), allocation concealment (selection bias), blinding of participants and personnel (performance bias), blinding of outcome assessors (detection bias), incomplete outcome data (attrition bias), selective outcome reporting (reporting bias), and other bias assessments. Three levels, namely, high, unclear, and low risk, were used to judge the risk bias of each study. Two of the authors (LZ and YT) performed the quality assessment and consulted with a third reviewer (MZ) when disagreements occurred.

### 2.7 Data synthesis and analysis

All the analyses were performed using RevMan5.2. Changes in continuous outcomes were calculated for each study arm by subtracting the value at baseline from the value after intervention. All the efficacy estimates were expressed as mean changes and 95% confidence interval (CI) from baseline. Standard deviations (SDs) were calculated from the standard error or 95% CI, according to the Cochrane Handbook for Systematic Review of Interventions. Safety estimates were presented as a pooled proportion with 95% CI. The Higgins *I* ^2^ statistics and Cochran’s Q test were used to assess the potential statistical heterogeneity among trials. I^2^ statistics more than 50% was considered heterogeneity. The meta-analysis was conducted using a random-effects model regardless of the *I* ^2^ value. Sensitivity analysis was conducted to assess the stability of the pooled effects. Dose–response of tirzepatide was undertaken according to the different doses. Subgroup analysis was carried out according to possible factors, leading to clinical heterogeneity, such as the background treatment, duration of T2D, and number of patients in each group < or ≥100. We also carried out an analysis comparing tirzepatide with GLP-1 RA. *p* < 0.05 was considered statistically significant. We used a funnel plot to judge the publication bias, which indicated no publication bias if the funnel plot was symmetrical; otherwise, there was a publication bias. Due to the subjectivity of the funnel plot, we also used Egger’s and Begg’s tests to verify the existence of publication bias through Stata (version 12, StataCorp). If the *p-*value of Egger’s or Begg’s test was less than 0.05, it indicated the presence of bias; otherwise, there was no bias.

## 3 Results

### 3.1 Search results

**FIGURE 1:**
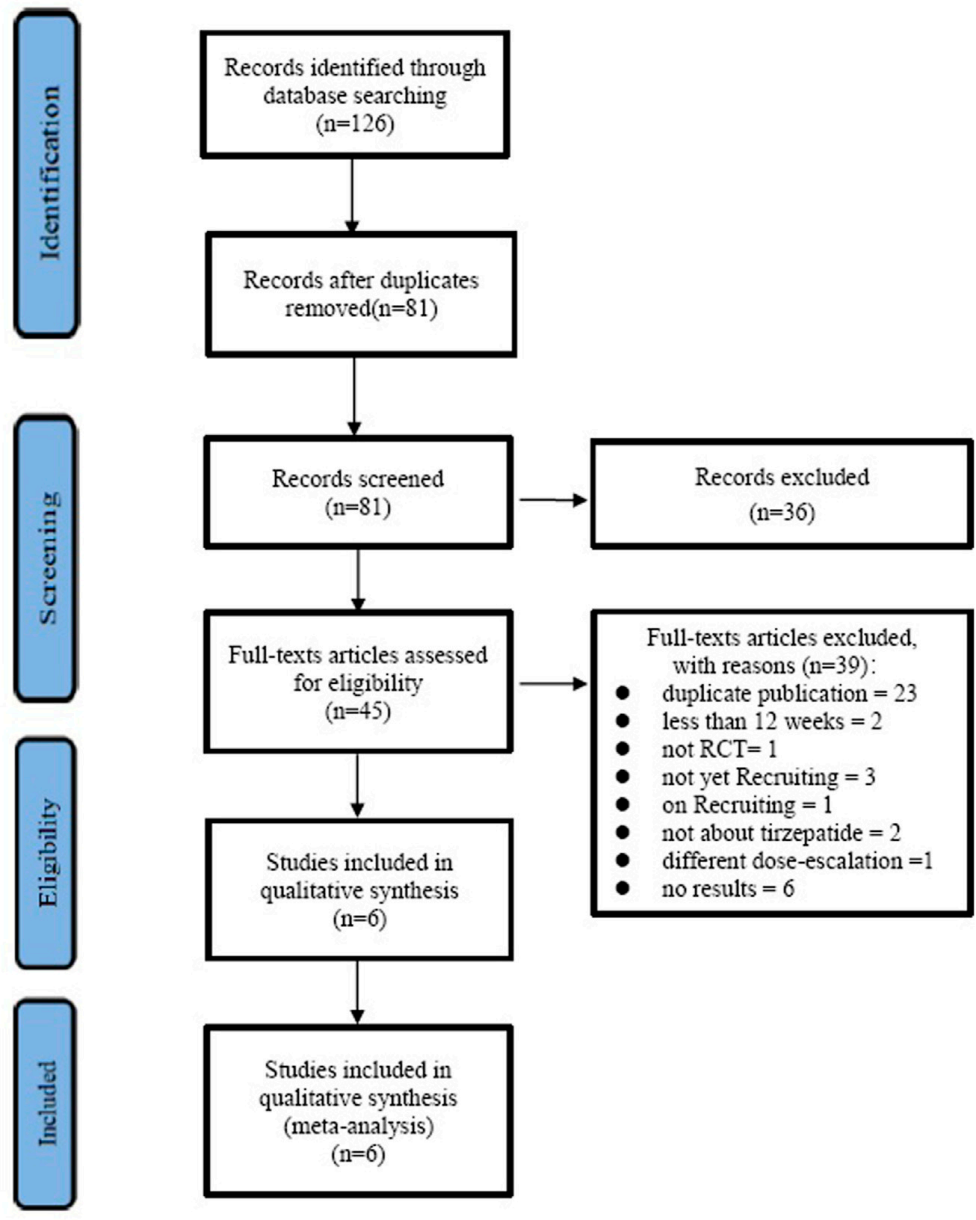
PRISMA flow diagram of the study.

The selection process is shown in Figure 1. With the search strategies, a total of 126 records were identified. Of these, 81 records were excluded because of duplication, 36 records were excluded according to titles and abstracts, and then 45 full articles were assessed for eligibility. Out of the 45 records, 23 were duplicates, two were less than 12 weeks, one was not RCT, three were not yet recruiting, one was on recruiting, two were not about tirzepatide, one was of a different dose-escalation, and six studies did not have results; the remaining six studies (Frias et al., 2018; Del Prato et al., 2021; Frias et al., 2021; Ludvik et al., 2021; Rosenstock et al., 2021; Dahl et al., 2022) satisfied the inclusion criteria and were included.

### 3.2 Study characteristics

The baseline characteristics of the study population are shown in Supplementary Table S1. The trials lasted from 26 to 52 weeks and compared tirzepatide with placebo (Frias et al., 2018; Rosenstock et al., 2021; Dahl et al., 2022), semaglutide 1 mg (Frias et al., 2021), insulin degludec (Ludvik et al., 2021), insulin glargine (Del Prato et al., 2021), and dulaglutide 1.5 mg (Frias et al., 2018). One trial was phase 2 (Frias et al., 2018) and the other five were phase 3 (Del Prato et al., 2021; Frias et al., 2021; Ludvik et al., 2021; Rosenstock et al., 2021; Dahl et al., 2022). There were a total of 4,410 and 2,052 patients with T2D in the tirzepatide group and the control group (171 in placebo, 469 in semaglutide 1 mg, 360 in insulin degludec, 1,000 in insulin glargine, and 54 in dulaglutide 1.5 mg), respectively. Three different levels of tirzepatide (5 mg, 10 mg, and 15 mg) were used in five studies (Del Prato et al., 2021; Frias et al., 2021; Ludvik et al., 2021; Rosenstock et al., 2021; Dahl et al., 2022). In one study (Frias et al., 2018), four levels of tirzepatide (1 mg, 5 mg, 10 mg, and 15 mg) were used. The mean HbA1c was from 7.85% to 8.59%, age was from 52.9 to 63.8 years, duration of T2D was from 3.7 to 14.1 years, and body weight was from 84.8 to 96.3 kg. Tirzepatide was used as monotherapy in one study (Rosenstock et al., 2021) and add-on therapy in the other five studies (Frias et al., 2018; Del Prato et al., 2021; Frias et al., 2021; Ludvik et al., 2021; Dahl et al., 2022). The background treatment was diet and exercise alone or with metformin in one study (Frias et al., 2018), with the proportion of participants using metformin being from 88.5% to 92.2%, which was considered an add-on therapy.

### 3.3 Risk of bias assessment

The Cochrane Collaboration’s Risk of Bias Tool including the risk-of-bias summary and risk-of-bias graph was used to assess the methodological quality, as shown in Supplementary Figures S1A and B. Random sequence generation, allocation concealment, blinding of participants and personnel, and blinding of outcome assessors were clearly presented in three studies (Frias et al., 2018; Rosenstock et al., 2021; Dahl et al., 2022). Random sequence generation and allocation concealment were unclear in two studies (Del Prato et al., 2021; Frias et al., 2021). Blinding of participants and personnel was not performed in three studies (Del Prato et al., 2021; Frias et al., 2021; Ludvik et al., 2021). The attrition bias, reporting bias, and other bias were low.

### 3.4 HbA1c

**FIGURE 2:**
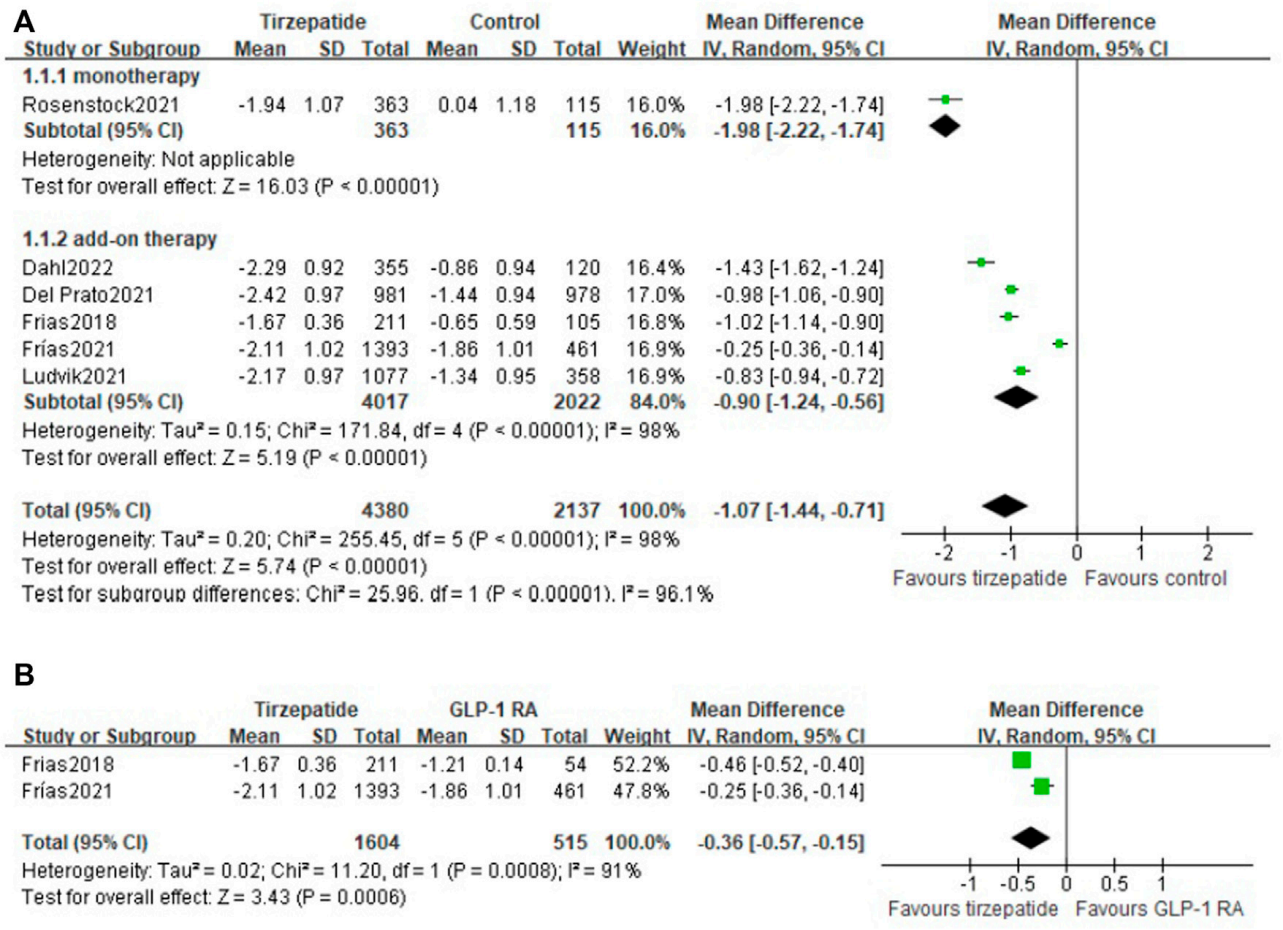
Weighted mean difference of the change in HbA1c from baseline (%): **(A)** tirzepatide vs. control and **(B)** tirzepatide vs. GLP-1 RA.

All the included studies reported the change in HbA1c from baseline. Compared with control, tirzepatide lowered HbA1c significantly (WMD, -1.07%; 95% CI: -1.44, -0.56) (*I* ^2^ = 98%; *p* < 0.00001). Both tirzepatide monotherapy (WMD, -1.98%; 95% CI: -2.22, -1.74) and add-on therapy (WMD, -0.90%; 95% CI: -1.24, -0.56) reduced HbA1c markedly (Figure 2A). We also compared the efficacy of tirzepatide with GLP-1 RA and demonstrated that tirzepatide decreased HbA1c obviously (WMD, −0.36%; 95% CI: −0.57, −0.15) (Figure 2B).

### 3.5 Percentage of patients with HbA1c <7%, ≤6.5%, or <5.7%

All included studies described the proportion of patients who reached the HbA1c target of <7% and ≤6.5%, and five studies (Frias et al., 2018; Del Prato et al., 2021; Frias et al., 2021; Ludvik et al., 2021; Dahl et al., 2022) presented the percentage of patients with the HbA1c target of <5.7%. The percentage of patients who reached the HbA1c target of <7.0% (62.6% vs. 41.2%; RR, 1.87; 95% CI: 1.51, 2.33) (*I* ^2^ = 92%; *p* < 0.00001) (Supplementary Figure S3), ≤6.5% (56.2% vs. 27.6%; RR, 2.43; 95% CI: 1.81, 3.28) (*I* ^2^ = 92%; *p* < 0.00001) (Supplementary Figure S4), or <5.7% (27.5% vs. 6.0%; RR, 5.85; 95% CI: 1.74, 19.65) (*I* ^2^ = 97%; *p* < 0.00001) (Supplementary Figure S5) was higher in the tirzepatide group than in the control group.

The RR in tirzepatide monotherapy subgroups at target HbA1c <7% and ≤6.5% levels were 4.45 (Supplementary Figure S3) and 4.52 (Supplementary Figure S4), respectively. The RR in tirzepatide add-on therapy subgroups at three target HbA1c levels were 1.66 (Supplementary Figure S3), 2.17 (Supplementary Figure S4), and 5.85 (Supplementary Figure S5), respectively. Both monotherapy and add-on therapy had a greater proportion than the control group.

Compared with GLP-1 RA, tirzepatide treatment showed a similar proportion of HbA1c reaching the target value of <7.0% (Supplementary Figure S6) or <5.7% (Supplementary Figure S7), except ≤6.5% (Supplementary Figure S8).

### 3.6 FSG

All the included studies reported the change in FSG from baseline. Tirzepatide led to a significantly greater reduction in FSG of 21.50 mg/dl (95% CI: −34.44, −8.56) (*I* ^2^ = 98%; *p* < 0.00001) than that in control, whether used as monotherapy (WMD, −59.17 mg/dl; 95% CI: −67.95, −50.39) or add-on therapy (WMD, −13.54 ml/dl; 95% CI: −22.78, −4.30) (Supplementary Figure S9). When compared with GLP-1 RA, tirzepatide decreased FSG obviously (WMD, −13.00 mg/dl; 95% CI: −18.90, −7.10) (Supplementary Figure S10).

### 3.7 Body weight

All the included studies reported a change in body weight from baseline.

Change in body weight was from −7.25 kg to −10.36 kg in the tirzepatide group and from 2.3 kg to -5.7 kg in the control group, and the mean treatment differences *versus* control were from -3.66 kg to -12.66 kg.

**FIGURE 3:**
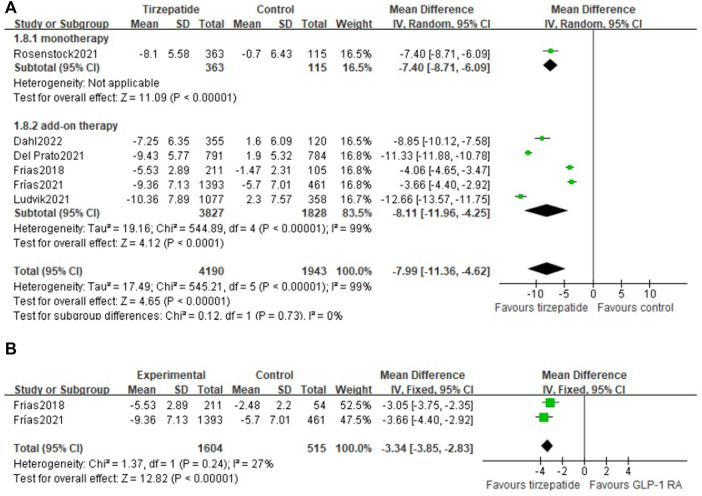
Weighted mean difference of the change in body weight from baseline (Kg): **(A)** tirzepatide vs. control and **(B)** tirzepatide vs. GLP-1 RA.

Compared with the control group, tirzepatide lowered body weight significantly (WMD: −7.99 kg; 95% CI: −11.36, −4.62), whether used as monotherapy or add-on therapy (monotherapy vs. control: WMD: −7.40 kg; 95% CI: −8.71, −6.09; add-on therapy vs. control: WMD: −8.11 kg; 95% CI: −11.96, −4.25) (Figure 3A). Tirzepatide reduced body weight more obviously than GLP-1 RA (WMD: −3.34 kg; 95% CI: −3.85, −2.83) (Figure 3B).

### 3.8 Number of patients with body weight ≥5%, ≥10%, or ≥15%

All studies described the number of patients who reached the body weight of ≥5%, ≥10%, or ≥15%. The percentage of patients who reached the three targets was higher in the tirzepatide group than in the control group. Both monotherapy and add-on therapy had a greater proportion of the three target body weight levels.

Compared with the control group, the RR in tirzepatide monotherapy subgroups at the three target body weight levels were 5.02, 37.38, and 36.65, respectively (Supplementary Figures S11–S13).

The RR in tirzepatide add-on therapy subgroups at the three levels in the number of patients with body weight was 5.80, 11.33, and 23.86, respectively (Supplementary Figures S11–S13).

Compared with GLP-1 RA, tirzepatide treatment showed a benefit in the proportion of body weight reaching ≥5% (Supplementary Figure S14), ≥10% (Supplementary Figure S15), and ≥15% (Supplementary Figure S16).

### 3.9 Blood pressure

Five studies (Frias et al., 2018; Del Prato et al., 2021; Ludvik et al., 2021; Dahl et al., 2022) reported a change in blood pressure. Compared with the control group, tirzepatide lowered systolic blood pressure and diastolic blood pressure significantly, whether used as tirzepatide monotherapy or add-on therapy (Supplementary Figures S17 and S18).

### 3.10 Fasting lipid profile

Four studies (Frias et al., 2018; Del Prato et al., 2021; Ludvik et al., 2021; Rosenstock et al., 2021) provided a percent change in the fasting lipid profile from baseline. Compared with the control group, tirzepatide lowered the percentage change of total cholesterol and triglycerides and increased HDL cholesterol significantly, whether used as monotherapy or add-on therapy (Supplementary Figures S19–S21).

### 3.11 MACE-4

Two studies (Del Prato et al., 2021; Dahl et al., 2022) evaluated the events of MACE-4. There were no differences between the tirzepatide and control groups (3.63% vs. 5.63%; RR, 0.76; 95% CI: 0.53, 1.09) (Supplementary Figure S22).

### 3.12 Adverse events

#### 3.12.1 Adverse events leading to treatment discontinuation

**FIGURE 4:**
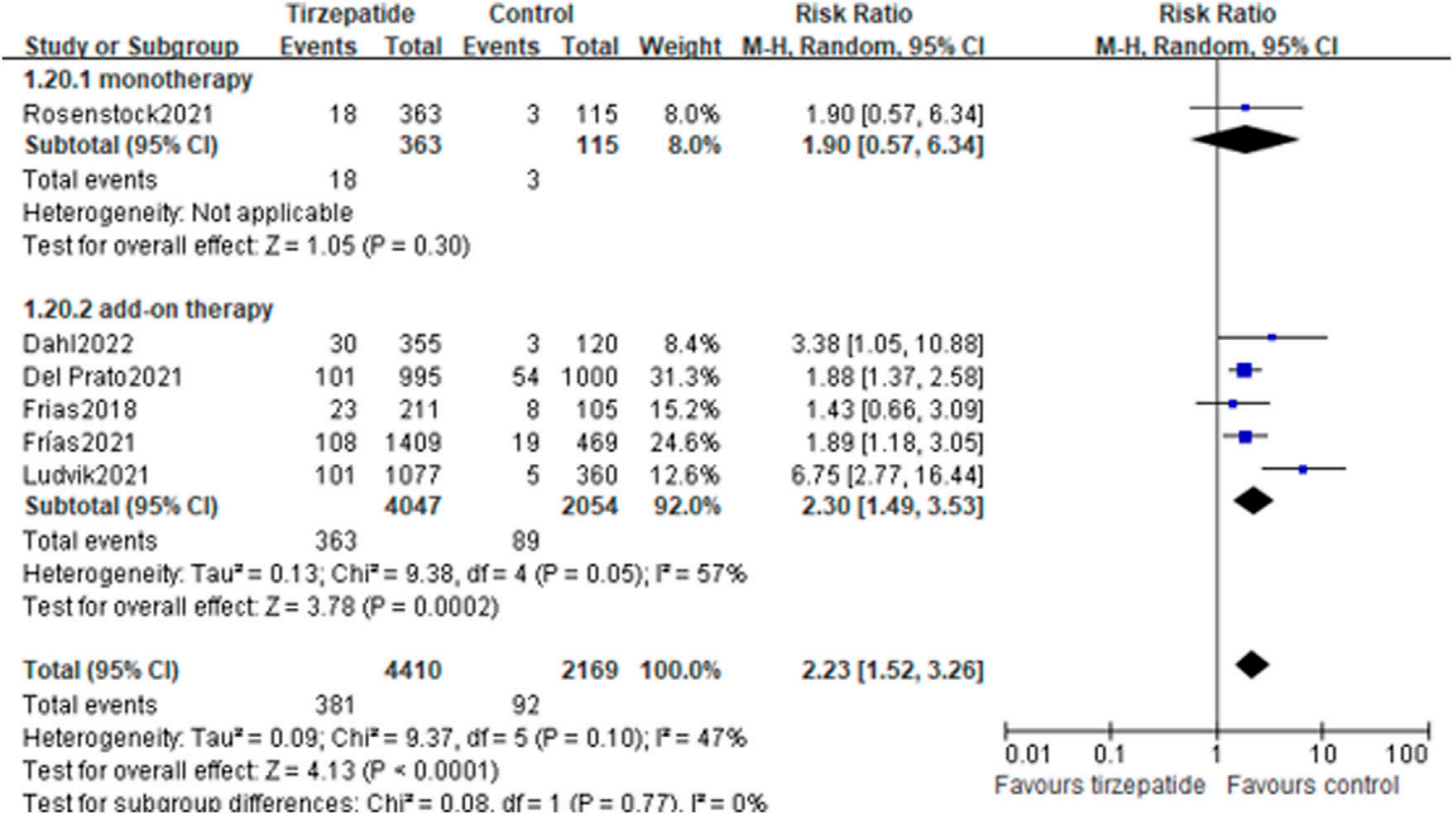
Risk ratio of adverse events leading to treatment discontinuation: tirzepatide vs. control.

Compared with the control group, tirzepatide increased the risk rate of adverse events leading to treatment discontinuation (8.64% vs. 4.24%; RR, 2.23; 95% CI: 1.52, 3.26) (Figure 4). Tirzepatide also showed an increase in this risk rate when compared with GLP-1 RA (8.09% vs. 4.78%; RR, 1.72; 95% CI: 1.11, 2.68) (Supplementary Figure S23).

#### 3.12.2 Hypoglycemia

**FIGURE 5:**
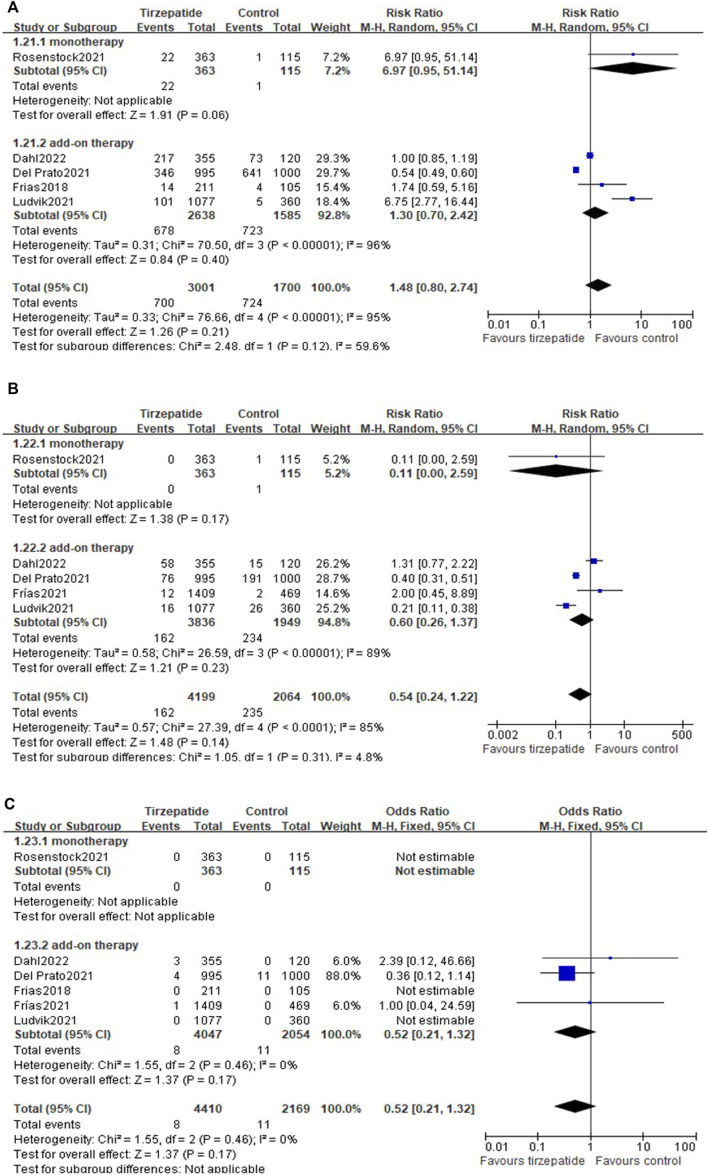
(Continued).

All the included studies reported the hypoglycemia events. Compared with the control group, tirzepatide did not increase the events of blood glucose <70 mg/dl (25.70% vs. 45.62%; RR, 1.48; 95% CI: 0.80, 2.74) (Figure 5A), <54 mg/dl (3.86% vs. 11.39%; RR, 0.54; 95% CI: 0.24, 1.22) (Figure 5B), or severe hypoglycemia (0.18% vs. 0.51%; RR, 0.52; 95% CI: 0.21, 1.32) (Figure 5C), whether used as monotherapy or add-on therapy (Figure 5).

Compared with GLP-1 RA, tirzepatide did not increase the events of blood glucose <70 mg/dl (Supplementary Figure S24), <54 mg/dl (Supplementary Figure S25), or severe hypoglycemia (Supplementary Figure S26).

#### 3.12.3 Gastrointestinal adverse reactions

**FIGURE 6:**
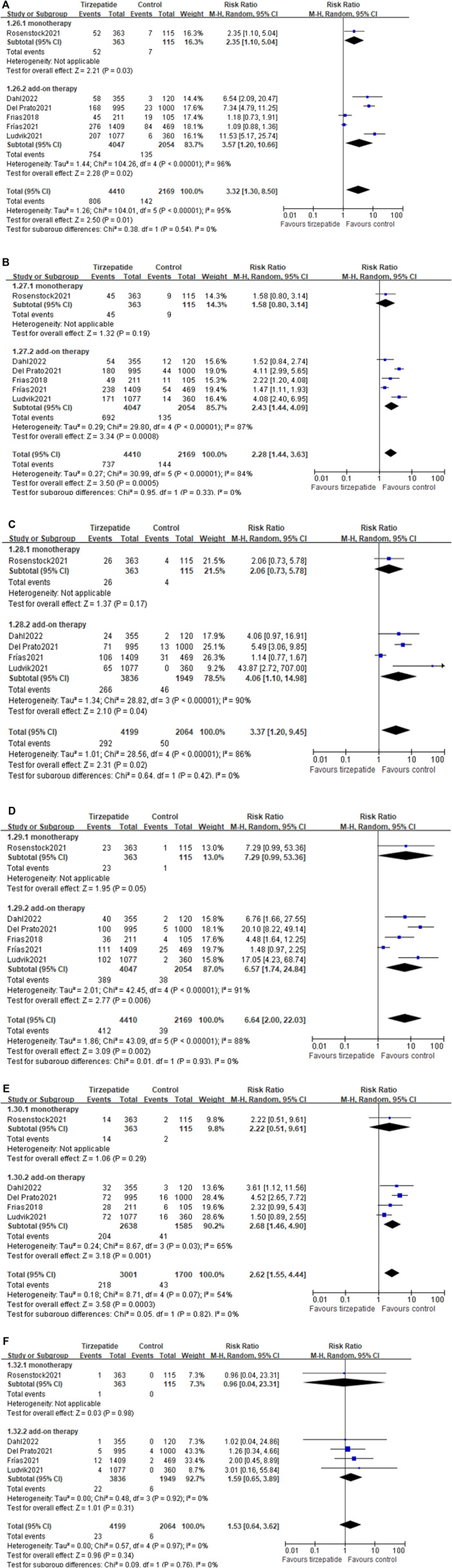
(Continued).

Compared with control, tirzepatide increased the risk ratio in nausea, diarrhea, dyspepsia, decreased appetite, and vomiting, but not cholelithiasis (Figure 6). Tirzepatide as add-on therapy increased gastrointestinal adverse events, while as monotherapy hardly increased gastrointestinal events, except nausea (Figure 6).

Compared with GLP-1 RA, there were no differences in the events of nausea, dyspepsia, decreased appetite, vomiting, or cholelithiasis (Supplementary Figures S27–S31), but differences were found in the event of increased diarrhea (Supplementary Figure S32).

#### 3.12.4 Pancreatitis

There were no differences in pancreatitis between tirzepatide and control (Supplementary Figure S33) and between tirzepatide and GLP-1 RA (Supplementary Figure S34).

### 3.13 Tirzepatide vs. control dose–response

Compared with control, four different levels (1 mg, 5 mg, 10 mg, and 15 mg) of tirzepatide presented a dose–response change from baseline in HbA1c (*p* < 0.00001), body weight (*p* < 0.00001), nausea (*p* = 0.004), and vomiting (*p* = 0.03) (Supplementary Figures S35–S38), but not in adverse events leading to treatment discontinuation (*p* = 0.14), blood glucose <70 mg/dl (*p* = 0.98), blood glucose <54 mg/dl (*p* = 0.82), severe hypoglycemia (*p* = 0.87), decreased appetite (*p* = 0.21), diarrhea (*p* = 0.46), pancreatitis (*p* = 0.83), and cholelithiasis (*p* = 0.79) (Supplementary Figures S39–S46).

### 3.14 Sensitivity analyses

Sensitivity analysis was employed in HbA1c, fasting plasma glucose, and body weight reduction by excluding each study one by one; there were no differences found in the changes, which showed the stability of the results.

### 3.15 Subgroup analysis

Due to the obvious heterogeneity of pooled data in HbA1c, we performed subgroup analysis based on monotherapy or add-on therapy (Supplementary Figure S47), duration of diabetes <10 years or ≥10 years (Supplementary Figure S48), and number of patients <100 or ≥100 (Supplementary Figure S49). We found that when the background treatment was divided into monotherapy or add-on therapy, heterogeneity also existed. When the subgroup analysis was based on the duration of diabetes or the number of patients, there was no heterogeneity. We believed that the source of heterogeneity may be related to these two factors.

### 3.16 Publication bias

The funnel plot is shown in Supplementary Figure S50 and seemed to be symmetrical. When Begg’s funnel (Supplementary Figure S51) and Begg’s and Egger’s tests were performed, the *p-*value was more than 0.05, which indicated that there was no publication bias.

## Discussion

Our meta-analysis, using surrogate metabolic endpoints, evaluated the comparative efficacy and safety of tirzepatide in patients with T2D insufficiently controlled by diet and exercise or other antihyperglycemic agents, including metformin, sulfonylurea, SGLT-2i, or insulin glargine. We found that, compared with control, tirzepatide reduced HbA1c, FSG, body weight, and blood pressure, ameliorated fasting lipid profiles, and increased the risk ratio of gastrointestinal adverse events (mainly as add-on therapy) but not in terms of MACE-4, hypoglycemia, pancreatitis, or cholelithiasis. We also observed that, compared with GLP-1 RA, tirzepatide treatment reduced HbA1c, FSG, and body weight and did not increase hypoglycemia and gastrointestinal adverse events except diarrhea. Tirzepatide treatment also presented a dose–response effect on reducing HbA1c and body weight and increasing nausea and vomiting, but not in terms of hypoglycemia, decreased appetite, diarrhea, pancreatitis, or cholelithiasis.

As for efficacy, our meta-analysis found that tirzepatide exhibited superior characteristics of lowering HbA1c by 1.07% and FSG by 21.50 mg/dl and had a greater proportion of participants reaching HbA1c reductions of 7%, 6.5% or greater, and 5.7%, both as monotherapy and add-on therapy. A meta-analysis including four RCTs showed that tirzepatide treatment lowered HbA1c and fasting glucose (Bhagavathula et al., 2021). The other meta-analysis including six RCTs and tirzepatide 12 mg indicated that tirzepatide had a greater reduction of HbA1c by 0.75% and FSG by 0.75 mmol/L (Dutta et al., 2021). The results were in good agreement with our results. However, we conducted a more in-depth study; according to whether tirzepatide combined with other hypoglycemic drugs or not, we divided tirzepatide treatment into monotherapy and add-on groups and further carried out the dose–response analysis. The results demonstrated that both tirzepatide as monotherapy and add-on therapy can reduce HbA1c and FSG significantly and showed a dose–response (1 mg, 5 mg, 10 mg, and 15 mg) effect on reducing HbA1c. These three doses (5 mg, 10 mg, and 15 mg) were more frequently used in clinical trials (Del Prato et al., 2021; Dahl et al., 2022) and may be chosen to use in clinics. However, the suitable clinical dose might differ between Asians and non-Asians, and clinical studies are needed to clarify this issue. GLP-1 RAs are a new class of antihyperglycemic drugs and are recommended by guidelines (Cosentino et al., 2020; American Diabetes Association Professional Practice Committee, 2022b). GLP-1 RAs as monotherapy can reduce HbA1c levels by 0.7–1.51%. Combined with other oral hypoglycemic drugs or as a part of triple therapy, the level of HbA1c can be further reduced by 0.4–1.9% (Klen and Dolzan, 2022). Our results demonstrated that tirzepatide decreased HbA1c by 0.36% and FSG by 13.00 mg/dl when compared with GLP-1 RAs (semaglutide 1 mg and dulaglutide 1.5 mg), which showed that tirzepatide had robust potential capabilities in glycemic control. At the same time, we also evaluated its performance in real-world patients whose characteristics are not completely consistent with RCTs (Zhou et al., 2021).

Obesity is an important concern in the management of type 2 diabetes due to body weight; weight management can postpone the development from prediabetes to T2D (Knowler et al., 2002) and bring benefits for the management of type 2 diabetes such as blood glucose and insulin resistance reduction (Rubino et al., 2016). For patients with T2D who are overweight or obese, moderate weight loss can result in the improvement of blood glucose control and reduce the demand for hypoglycemic drugs. Thus, when glucose-lowering agents are prescribed for overweight or obese patients with T2D, the effect on weight should be considered (American Diabetes Association Professional Practice Committee, 2022a). Clinically meaningful weight loss is typically defined as a decrease of at least >5% of usual body weight (American College of Cardiology/American Heart Association Task Force on Practice Guidelines, 2014). Our meta-analysis results demonstrated that loss of body weight in the tirzepatide group was from 7.25 kg to 10.36 kg, whether used as monotherapy (7.40 kg) or add-on therapy (8.11 kg). Tirzepatide therapy led to a greater target of the percentage of patients who reached the body weight reduction of ≥5%, ≥10%, and ≥15%, whether used as monotherapy or add-on therapy. More interestingly, weight loss was observed at all doses of tirzepatide within 4 weeks after the start of treatment, and this continued until week 40 or 52; no dose of tirzepatide reached a plateau (Ludvik et al., 2021; Rosenstock et al., 2021). The effect of tirzepatide on weight loss is expected. The subpopulation of the SURPASS-3 study showed that tirzepatide led to a significant reduction in liver fat content, volume of visceral adipose tissue, and abdominal subcutaneous adipose tissue volumes compared with insulin degludec (Gastaldelli et al., 2022).

GLP-1 RAs not only show a good hypoglycemic effect but also demonstrate the efficacy of weight loss. A meta-analysis evaluated the weight reduction effects of GLP-1 RAs and exhibited that, compared to placebo, GLP-1 RAs led to significant body weight reduction (Htike et al., 2017). As for semaglutide administered in patients with T2D, compared with placebo, subcutaneous semaglutide led to body weight loss (WMD: -2.73 kg and -4.09 kg, for 0.5 mg and 1 mg, respectively). Oral administration showed similar effects (Zhong et al., 2021). For treatment with dulaglutide in patients with T2D, the mean weight loss was 0.73 kg in the 0.75 mg dulaglutide group and 1.27 kg in the 1.5 mg dulaglutide group (Qie et al., 2020). Our meta-analysis included semaglutide 1 mg and dulaglutide 1.5 mg as the control and presented that tirzepatide reduced body weight greater than the two GLP-1 RAs and resulted in a higher proportion of body weight reaching ≥5%, ≥10%, and ≥15%.

Blood pressure and lipid profiles are the risk factors of cardiovascular events. Tirzepatide intervention can result in favorable changes in lowering blood pressure and fasting lipoprotein profiles, including reductions in total cholesterol and triglyceride and increase in HDL cholesterol, whether used as monotherapy or add-on therapy. As for GLP-1 RAs, most of them lowered SBP notably but had no significant effect on DBP and blood lipid outcomes (Jiang et al., 2021).

At present, cardiovascular safety is an important index to consider for hypoglycemic drugs. Our result found no differences between tirzepatide and control, which indicated that tirzepatide did not increase MACE-4 and showed cardiovascular safety. However, liraglutide, semaglutide, and dulaglutide administered to people with T2D resulted in a significant decrease in MACE (Marso et al., 2016; Mann et al., 2017; Gerstein et al., 2019). A meta-analysis focused on the effect of GLP-1 RA on cardiovascular outcomes and demonstrated that GLP-1 RA had moderate benefits on MACE (Giugliano et al., 2021). A pre-specified meta-analysis indicated that tirzepatide did not increase the risk of major cardiovascular events in T2D participants compared with the control group (Sattar et al., 2022). The results may be confirmed by the SURPASS-CVOT ongoing study (NCT04255433), which compares tirzepatide with dulaglutide in patients with T2D and high risk for MACE.

Hypoglycemia is the main challenge in achieving a target HbA1c of less than 7% in patients with T2D. Tirzepatide showed improvements in blood glucose control and, at the same time, did not increase the risk of mild-to-moderate and severe hypoglycemia, both when used as monotherapy and add-on therapy. The rate of blood glucose <70 mg/dl and <54 mg/dl was 34.77% and 7.64% in SURPASS-4 (Del Prato et al., 2021) and 61.13% and 16.34% in SURPASS-5 (Dahl et al., 2022), respectively, which appeared to be higher. The use of sulfonylurea as a part of the background treatment in SURPASS-4 and the use of insulin glargine in SURPASS-5 were probably the reason. When hypoglycemia was considered, GLP-1 RA was referred to be semaglutide 1 mg in our meta-analysis. Rates in patients with hypoglycemia who received semaglutide 0.5 mg and 1 mg were 23.1% and 21.7%, respectively, which was similar to those of the placebo (21.2%) in the SUSTAIN-6 study (Marso et al., 2016). There were no differences in hypoglycemia between tirzepatide and semaglutide 1 mg. In a word, the risk of hypoglycemia seems to be low for subcutaneous tirzepatide; however, it increases when used in combination with sulfonylureas or insulin therapy.

Gastrointestinal adverse reactions are common side effects of GLP-1 RAs. GLP-1 RA can not only bind to the GLP-1 receptor of the gastrointestinal tract and inhibit gastric emptying but also aggravate anorexia and/or satiety by activating central GLP-1 receptors, which are widely distributed in the brain (Shah and Vella, 2014). Tirzepatide, a fatty acid-modified double intestinal insulinotropic receptor agonist, shows similar pharmacology to natural GIP on glucose-dependent insulinotropic polypeptide receptor (GIPR) but shows a preference for the cyclic adenosine monophosphate signal on GLP-1R (Sun B. et al., 2022a). Similar to GLP-1 RAs, compared with control, tirzepatide increases the risk of gastrointestinal adverse reactions, however, mainly due to add-on therapy. Monotherapy hardly increased gastrointestinal events. The incidence of gastrointestinal adverse events was similar between tirzepatide and GLP-1 RAs; however, it increased diarrhea risk.

Acute pancreatitis is an event of concern during the use of GLP-RAs. At present, there is no enough evidence suggesting an increase in the risk of pancreatitis with the use of GLP-1 RA (Storgaard et al., 2017). Tirzepatide did not show an increase in the risk of pancreatitis. The dose–response effect for drugs is an important consideration in the clinical decision in both efficacy and safety aspects. This review has revealed the dose–response effect of tirzepatide on the treatment of T2D with the dose of 1 mg–15 mg, which indicates that high doses possessed higher efficacy for blood glucose control and body weight reduction, without mild-to-severe hypoglycemia or some gastrointestinal adverse events, than the lower doses. The dose–response analysis provided additional information on the choice of tirzepatide, whether to be used as monotherapy or add-on therapy.

Our present meta-analysis has certain limitations. First, there is obvious heterogeneity in the included studies, which may influence the strength of the results, though the random effect was used. Second, the trial duration was from 26 to 52 weeks, which was not enough to evaluate the hard endpoints, such as cardiovascular events and all-cause death. Third, although there was no publication bias, the *p*-value of Begg’s test and Egger’s test was 0.06 and 0.091, respectively, and the funnel plot did not seem to be symmetric, which suggested that we should pay attention to the possible publication bias.

## Conclusion

In conclusion, our systematic review and meta-analysis provides comprehensive estimates of the effects of tirzepatide on T2D. Tirzepatide treatment resulted in beneficial effects in terms of HbA1c, FSG, body weight, blood pressure, fasting lipid profiles, and HOMA2-IR, without increasing hypoglycemia, either as monotherapy or as an add-on therapy. Tirzepatide increased the risk ratio of gastrointestinal adverse events mainly in add-on therapy but not in terms of MACE-4, pancreatitis, or cholelithiasis. Furthermore, tirzepatide treatment presented a dose–response effect on HbA1c control, body weight reduction, and nausea and vomiting increase but not on hypoglycemia, decreased appetite, diarrhea, pancreatitis, or cholelithiasis. Additional long-term studies for assessing the possibility of cardiovascular and renal protection in patients with T2D and anti-obesity effects on people with obesity without DM are needed.

## Data Availability

The original contributions presented in the study are included in the article/Supplementary materials; further inquiries can be directed to the corresponding author.
