# Supplementary material for: Efficacy and safety of tirzepatide in patients with type 2 diabetes: A systematic review and meta-analysis
Source: Front Pharmacol. 2022 Oct 28;13:1016639. doi: 10.3389/fphar.2022.1016639 (PMC9774036; doi:10.3389/fphar.2022.1016639)
Supplement: Supplementary file 1 [file Table1.DOC]

Table S1. Baseline characteristics of the study population included in the meta-analysis

| Primary study | Study duration（weeks） | Source of information | Study arms included in meta-analyses | No. of patients randomised | HbA1c  (%) | Age  (years) | Duration of T2DM（years） | Body Weight (kg) | Background treatment | Countries/regions |
| --- | --- | --- | --- | --- | --- | --- | --- | --- | --- | --- |
| Rosenstock  2021 | 40  Phase 3 | NCT03954834  (SURPASS-1) | placebo | 115 | 8.05 ± 0.80 | 53.6 ±12.8 | 4.5±5.9 | 84.8 ± 20.0 | diet and exercise,  no drugs | 52 medical research centres and hospitals in India, Japan, Mexico, and the USA |
|  |  |  | tirzepatide 5mg | 121 | 7.97± 0.84 | 54.1± 11.9 | 4.6±5.1 | 87.0 ± 21.2 |  |  |
|  |  |  | tirzepatide 10mg | 121 | 7.90 ± 0.78 | 55.8±10.4 | 4.9±5.6 | 86.2 ±19.5 |  |  |
|  |  |  | tirzepatide 15mg | 121 | 7.85 ± 1.02 | 52.9± 12.3 | 4.8±5.0 | 85.4 ± 18.5 |  |  |
| Frías2021 | 40  Phase 3 | NCT03987919  (SURPASS-2) | semaglutide 1mg | 469 | 8.25±1.01 | 56.9±10.8 | 3.9±4.7 | 93.7±21.12 | metformin | 122 medical research centres and hospitals in Argentina, Austria, Greece, Hungary, Italy, Poland, Puerto Rico, Romania, South Korea, Spain, Taiwan, Ukraine, and the USA. |
|  |  |  | tirzepatide 5mg | 470 | 8.32±1.08 | 56.3±10.0 | 3.9±3.2 | 92.5±21.76 |  |  |
|  |  |  | tirzepatide 10mg | 469 | 8.30±1.02 | 57.2±10.5 | 3.7±3.8 | 94.8±22.71 |  |  |
|  |  |  | tirzepatide 15mg | 470 | 8.26±1.00 | 55.9±10.4 | 4.6±4.1 | 93.8±21.83 |  |  |
| Ludvik2021 | 52  Phase 3 | NCT03882970  (SURPASS-3) | insulin degludec | 360 | 8.12 ±0.94 | 57.5±10.1 | 8.1±6.0 | 94.0±20.6 | metformin ± SGLT-2i | 122 medical research centres and hospitals in Argentina, Austria, Greece, Hungary, Italy, Poland, Puerto Rico, Romania, South Korea, Spain, Taiwan, Ukraine, and the USA. |
|  |  |  | tirzepatide 5mg | 358 | 8.17 ± 0.89 | 57.2±10.1 | 8.5±5.8 | 94.4±18.9 |  |  |
|  |  |  | tirzepatide 10mg | 360 | 8.18± 0.89 | 57.4±9.7 | 8.4±6.6 | 93.8±19.8 |  |  |
|  |  |  | tirzepatide 15mg | 359 | 8.21 ±0.94 | 57.5±10.2 | 8.5±6.5 | 94.9±21.0 |  |  |
| Del Prato2021 | 52  phase 3 | NCT03730662  (SURPASS-4) | insulin glargine | 1000 | 8.50±0.85 | 63.8±8.5 | 10.7 (6.3-16.5)^$^ | 90.2±19.00 | with any of three oral glucose-lowering medications (ie, metformin, sulfonylurea, or SGLT-2i ) either alone or in any combination | Argentina, Australia, Brazil, Canada, Greece, Israel, Mexico, Poland, Romania, Russia, Slovakia, Spain, Taiwan, and the USA |
|  |  |  | tirzepatide 5mg | 329 | 8.52±0.84 | 62.9±8.6 | 9.8 (6.2-15.3)^$^ | 90.3±20.32 |  |  |
|  |  |  | tirzepatide 10mg | 328 | 8.59±0.91 | 63.7±8.7 | 10.6 (6.5-16.2)^$^ | 90.6±18.21 |  |  |
|  |  |  | tirzepatide 15mg | 338 | 8.52±0.98 | 63.7±8.6 | 10.4 (5.5-15.7)^$^ | 90.0±16.34 |  |  |
| Dahl2022 | 40  phase 3 | NCT04039503  (SURPASS-5) | placebo | 120 | 8.37±0.84 | 60±10 | 12.9±7.4 | 94.1±21.8 | insulin glargine with or without metformin | 45 medical research centers and hospitals in the USA |
|  |  |  | tirzepatide 5mg | 116 | 8.30±0.88 | 62±10 | 14.1±8.1 | 95.8±19.8 |  |  |
|  |  |  | tirzepatide 10mg | 119 | 8.36±0.83 | 60±10 | 12.6±6.2 | 94.5±22.2 |  |  |
|  |  |  | tirzepatide 15mg | 120 | 8.23±0.86 | 61±10 | 13.7±7.5 | 96.3±22.8 |  |  |
| Frias2018 | 26  phase 2 | NCT03131687 | placebo | 51 | 8.0 ± 0.9 | 56.6±8.9 | 8.6±7.0 | 91.5±23.1 | diet and exercise alone or with metformin | 47 sites (medical and clinical research centres)  in Poland, Puerto Rico, Slovakia, US |
|  |  |  | tirzepatide 1mg | 52 | 8.2 ± 0.9 | 57.4±8.9 | 7.8±5.4 | 93.2±24.4 |  |  |
|  |  |  | tirzepatide 5mg | 55 | 8.2± 1.0 | 57.9±8.2 | 8.9±5.7 | 92.8±19.0 |  |  |
|  |  |  | tirzepatide 10mg | 51 | 8.2± 1.1 | 56.5±9.9 | 7.9±5.8 | 92.7±19.5 |  |  |
|  |  |  | tirzepatide 15mg | 53 | 8.1±1.0 | 56.0±7.6 | 8.5±6.1 | 89.1±22.7 |  |  |
|  |  |  | dulaglutide 1.5mg | 54 | 8.1±1.0 | 58.7±7.8 | 9.3±7.1 | 89.1±22.7 |  |  |

Data are mean (SD), or $, median (IQR). HbA_1c_=glycated haemoglobin. T2DM=type 2 diabetes mellitus. SGLT-2i=sodium -glucose co-transporter-2 inhibitor
