# Supplementary material for: Efficacy and safety of tirzepatide in patients with type 2 diabetes: A systematic review and meta-analysis
Source: Front Pharmacol. 2022 Oct 28;13:1016639. doi: 10.3389/fphar.2022.1016639 (PMC9774036; doi:10.3389/fphar.2022.1016639)
Supplement: Supplementary file 2 [file DataSheet1.DOCX]

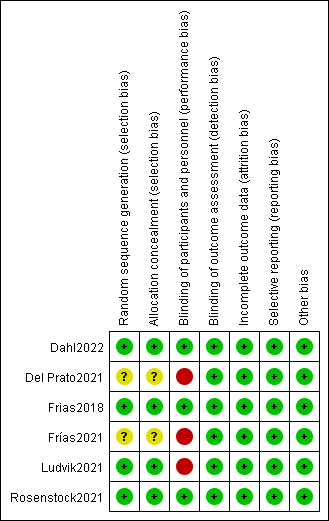


A．Risk of bias summary


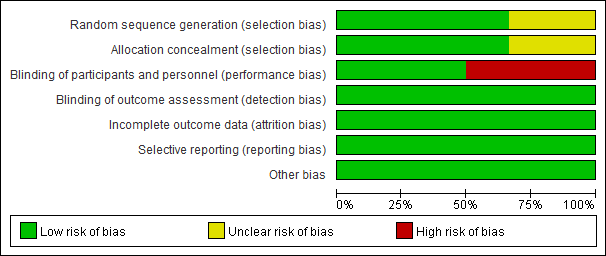


B．Risk of bias graph

Fig. S1: A.Risk of bias summary; B. risk of bias graph.


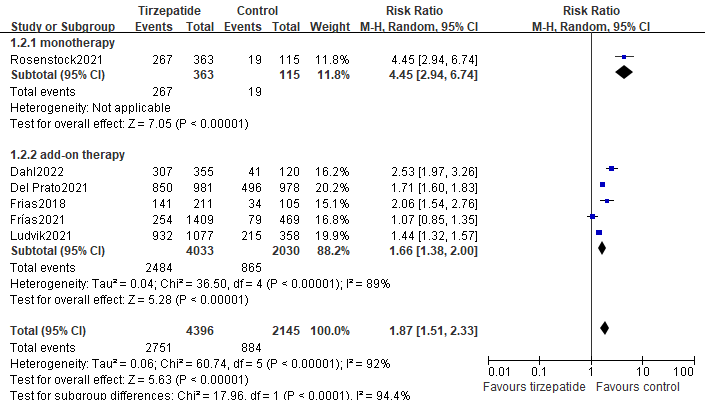


Supplementary Fig. S3 Risk ratio in change from baseline in percentage of patients with HbA1c <7%: tirzepatide *vs*. control.


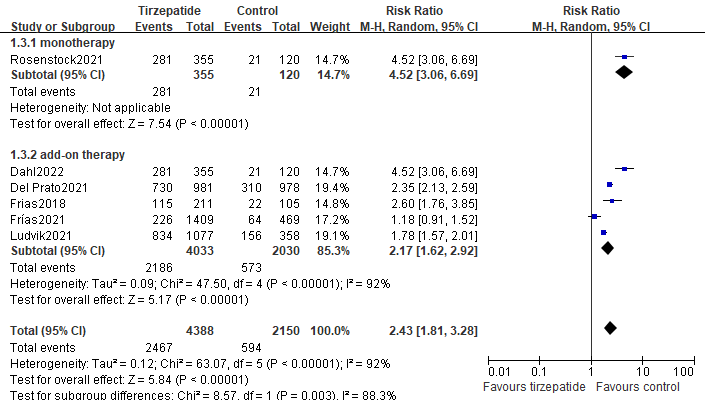


Supplementary Fig. S4 Risk ratio in change from baseline in percentage of patients with HbA1c ≤6.5%: tirzepatide *vs*. control.


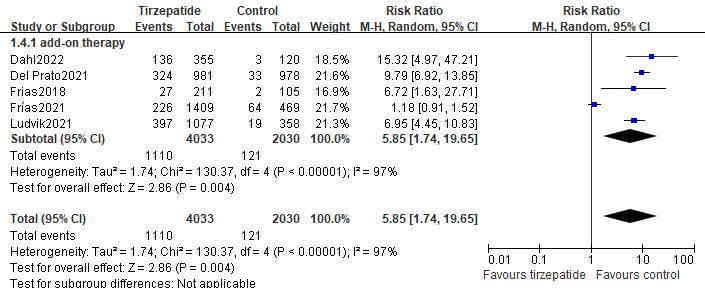


Supplementary Fig. S5 Risk ratio in change from baseline in percentage of patients with HbA1c <5.7%: tirzepatide *vs*. control.


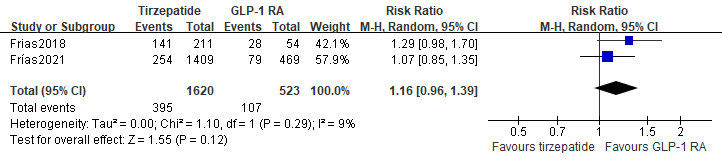


Supplementary Fig. S6 Risk ratio in change from baseline in percentage of patients with HbA1c <7.0%: tirzepatide *vs.* GLP-1 RA.


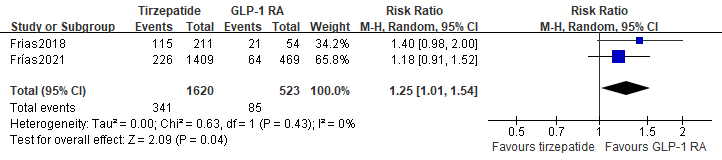


Supplementary Fig. S7 Risk ratio in change from baseline in percentage of patients with HbA1c ≤6.5%: tirzepatide *vs*. GLP-1 RA.


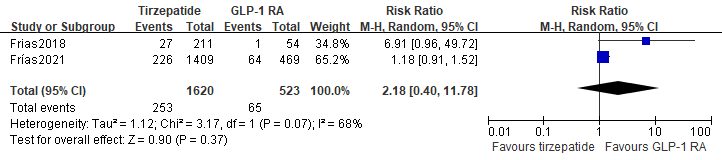


Supplementary Fig. S8 Risk ratio in change from baseline in percentage of patients with HbA1c <5.7%: tirzepatide *vs*. GLP-1 RA.


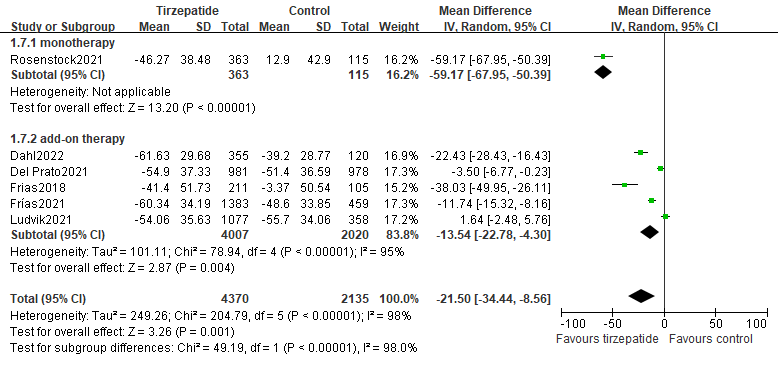


Supplementary Fig. S9 The weighted mean difference in change from baseline in FSG (mg/dL): tirzepatide *vs*. control.


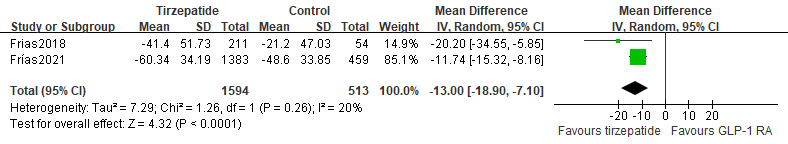


Supplementary Fig. S10 The weighted mean difference in change from baseline in FSG (mg/dL): tirzepatide *vs*. GLP-1 RA.


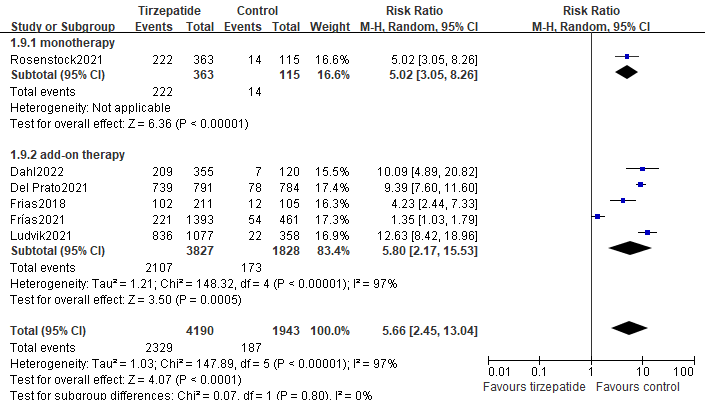


Supplementary Fig. S11 Risk ratio in change from baseline in percentage of patients with body weight ≥5%: tirzepatide *vs*. control.


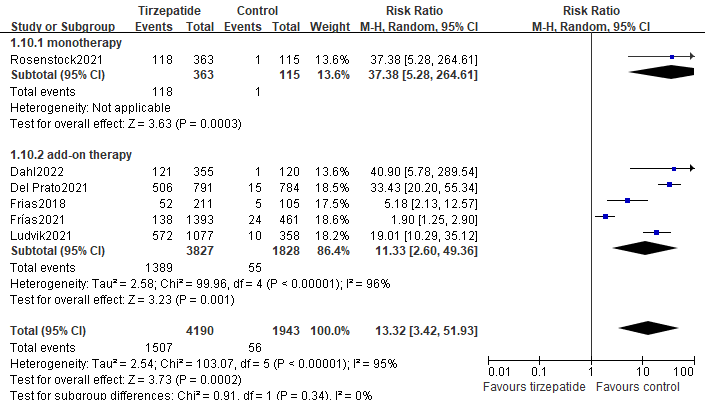


Supplementary Fig. S12 Risk ratio in change from baseline in percentage of patients with body weight≥10%: tirzepatide *vs*. control.


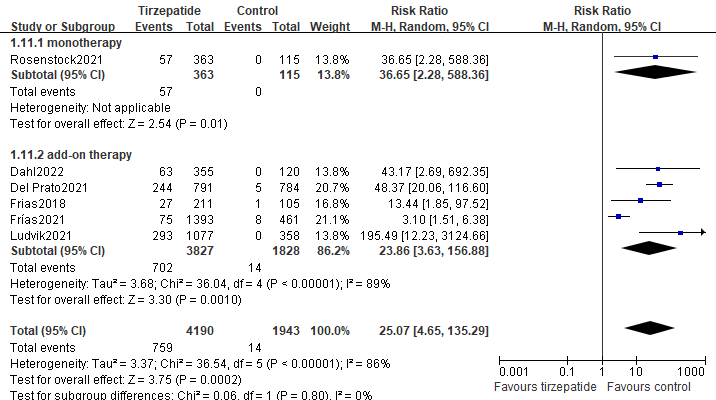


Supplementary Fig. S13 Risk ratio in change from baseline in percentage of patients with body weight≥15%: tirzepatide *vs*. control.


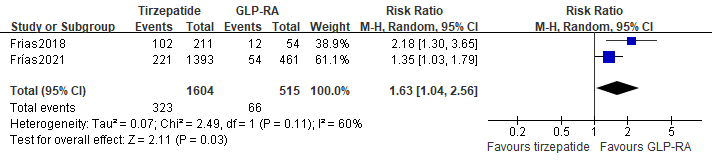


Supplementary Fig. S14 Risk ratio in change from baseline in percentage of patients with body weight ≥5%: tirzepatide *vs*. GLP-1 RA.


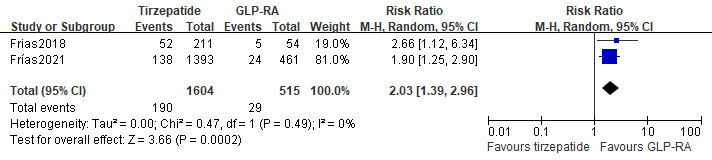


Supplementary Fig. S15 Risk ratio in change from baseline in percentage of patients with body weight ≥10%: tirzepatide *vs*. GLP-1 RA.


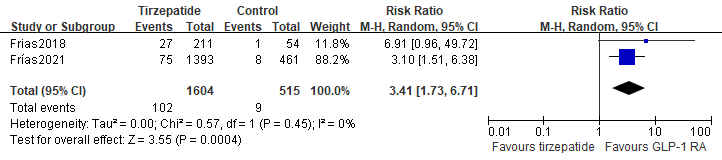


Supplementary Fig. S16 Risk ratio in change from baseline in percentage of patients with body weight ≥15%: tirzepatide *vs*. GLP-1 RA.


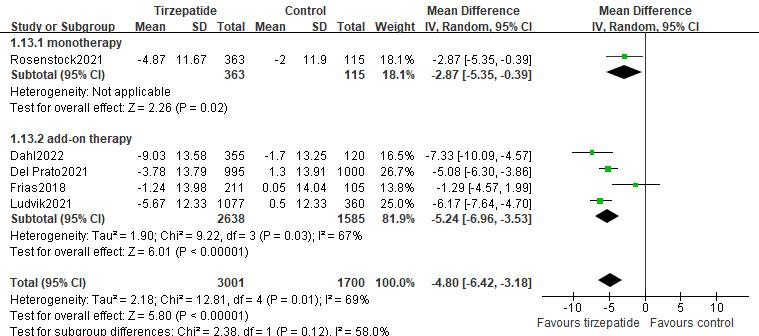


Supplementary Fig. S17 The weighted mean difference in change from baseline in systolic blood pressure (mmHg): tirzepatide *vs*. control.


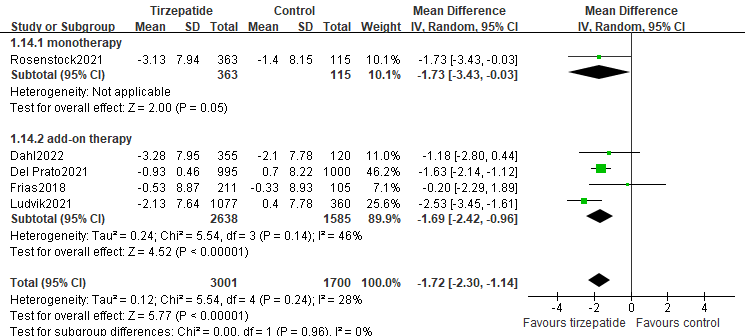


Supplementary Fig. S18 The weighted mean difference in change from baseline in diastolic blood pressure (mmHg): tirzepatide *vs*. control.


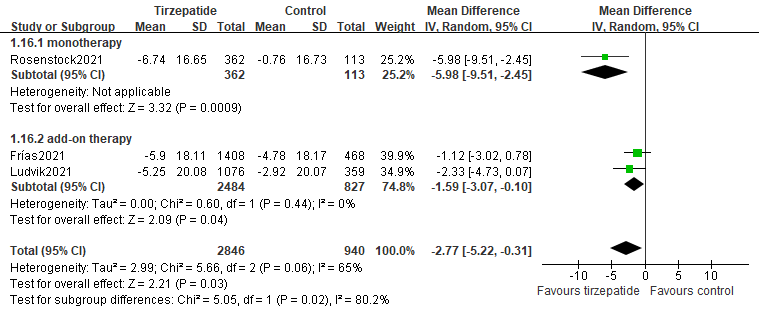


Supplementary Fig. S19 The weighted mean difference in change from baseline in percentage change of total cholesterol from baseline (%): tirzepatide *vs*. control.


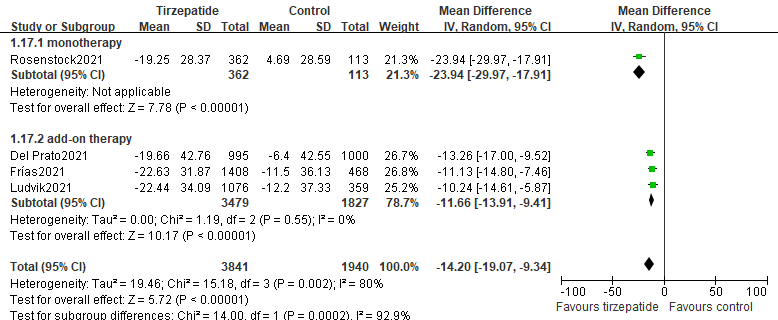


Supplementary Fig. S20The weighted mean difference in change from baseline in percentage change of triglycerides from baseline (%): tirzepatide *vs*. control.


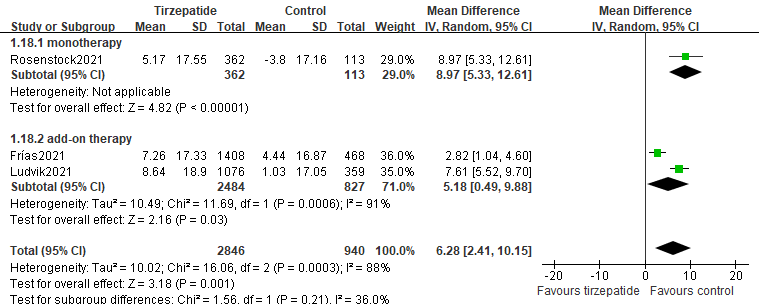


Supplementary Fig. S21 The weighted mean difference in change from baseline in percentage change of HDL cholesterol from baseline (%): tirzepatide *vs*. control.


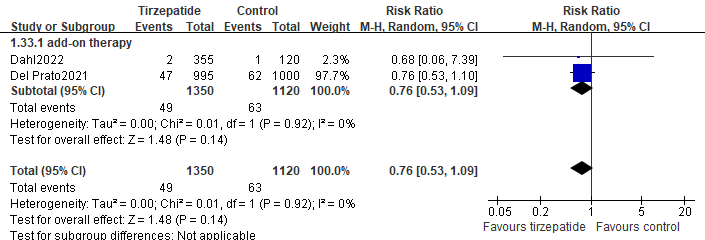


Supplementary Fig. S22 Risk ratio in MACE-4: tirzepatide *vs*. control.


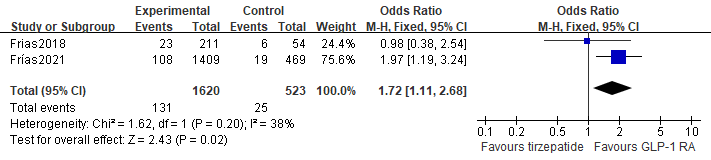


Supplementary Fig. S23 Risk ratio in adverse events leading to treatment discontinuation: tirzepatide *vs.* GLP-1 RA.


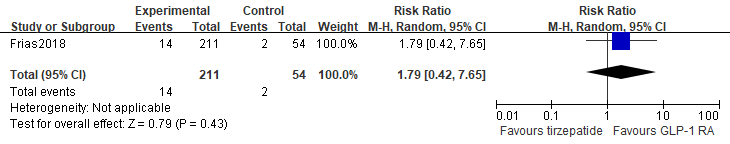


Supplementary Fig. S24 Risk ratio in hypoglycaemia (blood glucose <70 mg/dL): tirzepatide *vs*. GLP-1 RA.


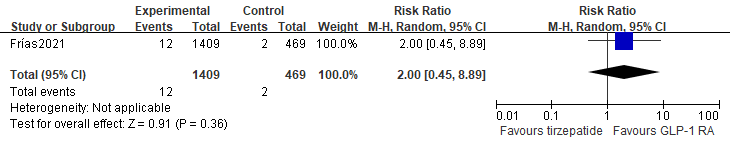


Supplementary Fig. S25 Risk ratio in hypoglycaemia (blood glucose <54mg/dL): tirzepatide *vs*. GLP-1 RA.


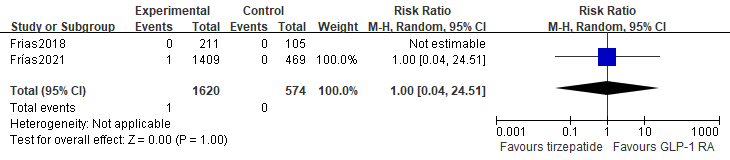


Supplementary Fig. S26 Risk ratio in severe hypoglycaemia: tirzepatide *vs*. GLP-1 RA.


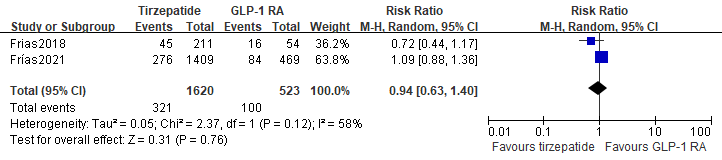


Supplementary Fig. S27 Risk ratio in nausea: tirzepatide *vs*. GLP-1 RA.


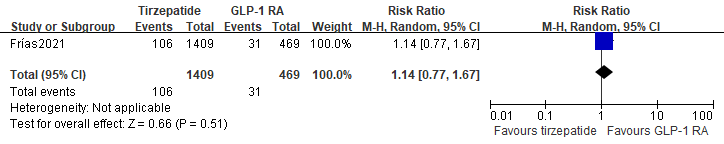


Supplementary Fig. S28 Risk ratio in dyspepsia: tirzepatide *vs*. GLP-1 RA.


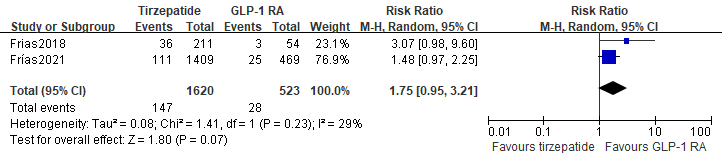


Supplementary Fig. S29Risk ratio in decreased appetite: tirzepatide *vs*. GLP-1 RA.


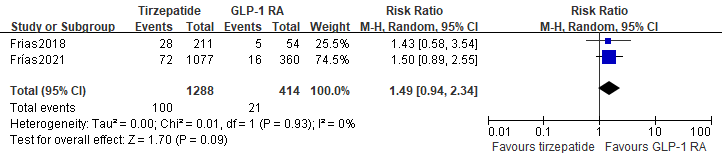


Supplementary Fig. S30 Risk ratio in vomiting: tirzepatide *vs.* GLP-1 RA.


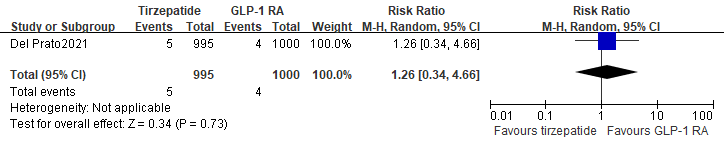


Supplementary Fig. S31 Risk ratio in cholelithiasis: tirzepatide *vs*. GLP-1 RA.


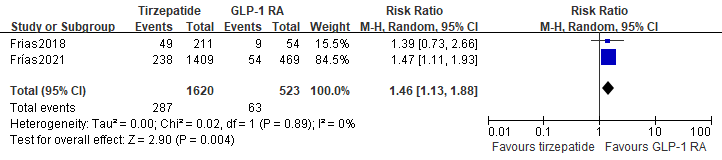


Supplementary Fig. S32 Risk ratio in diarrhoea: tirzepatide *vs.* GLP-1 RA.

**
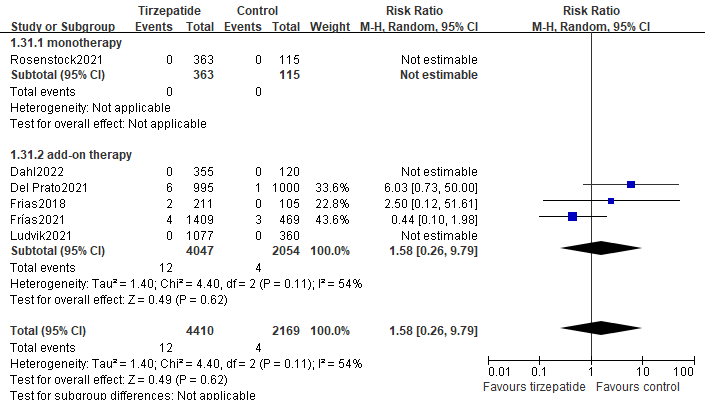
**

Supplementary Fig. S33 Risk ratio in pancreatitis: tirzepatide *vs*. control.


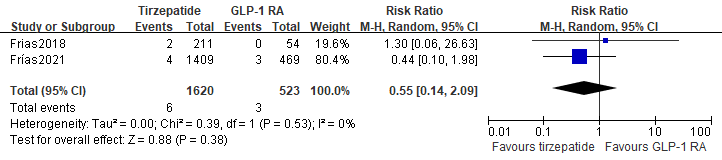


Supplementary Fig. S34 Risk ratio in pancreatitis: tirzepatide *vs*. GLP-1 RA.


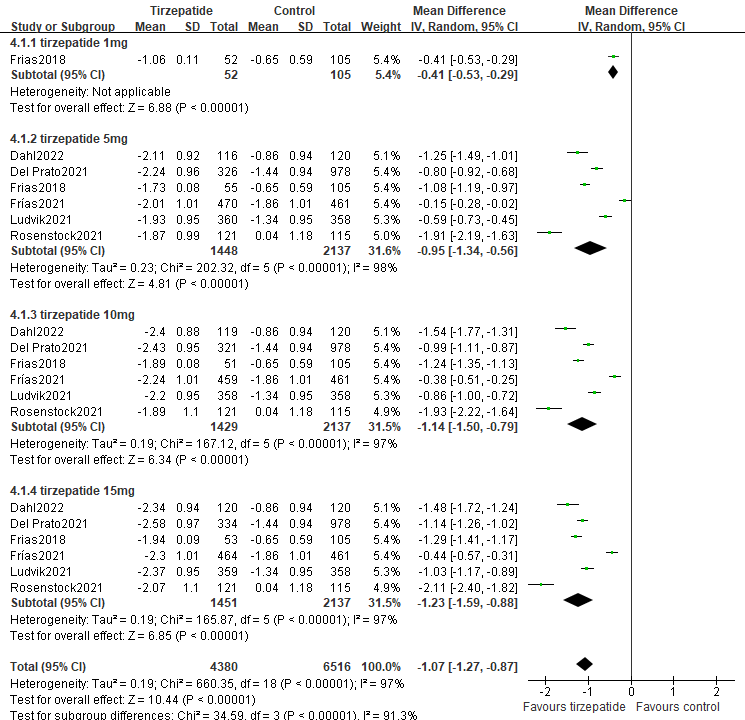


Supplementary Fig. S35 Dose-response change from baseline in HbA1c: tirzepatide *vs.* control


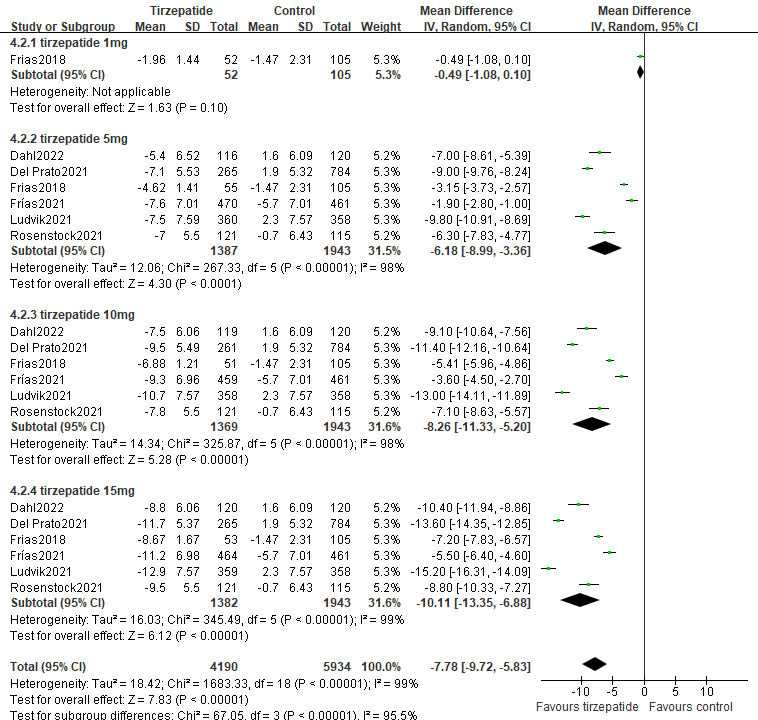


Supplementary Fig. S36 Dose-response of body weight change from baseline: tirzepatide *vs.* control
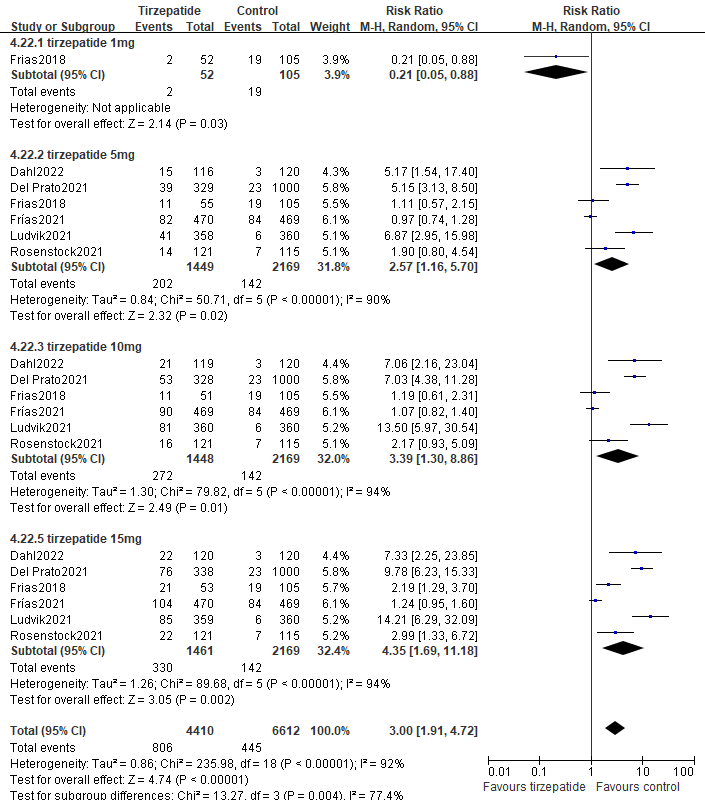
Supplementary Fig. S37 Dose-response of nausea: tirzepatide *vs*. control**
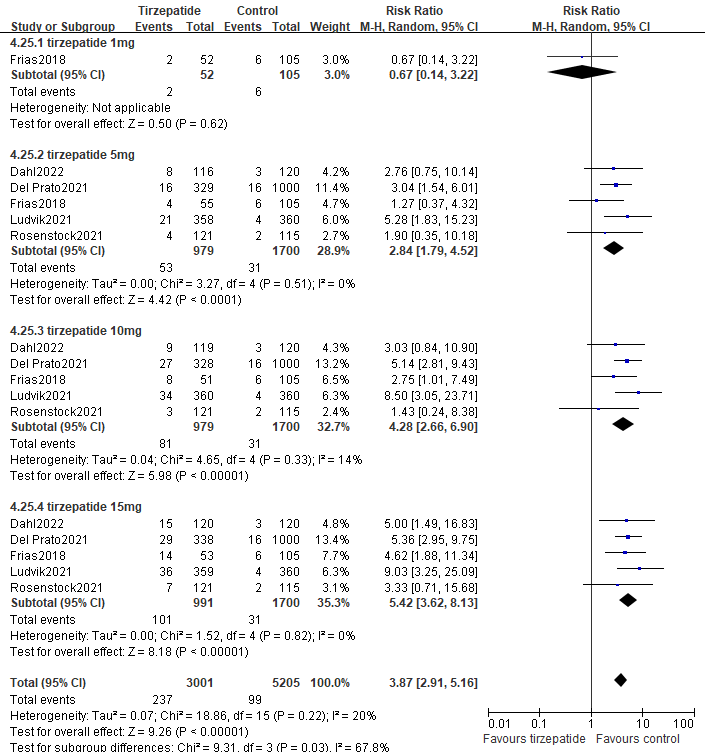
**

Supplementary Fig. S38 Dose-response of vomiting: tirzepatide *vs*. control


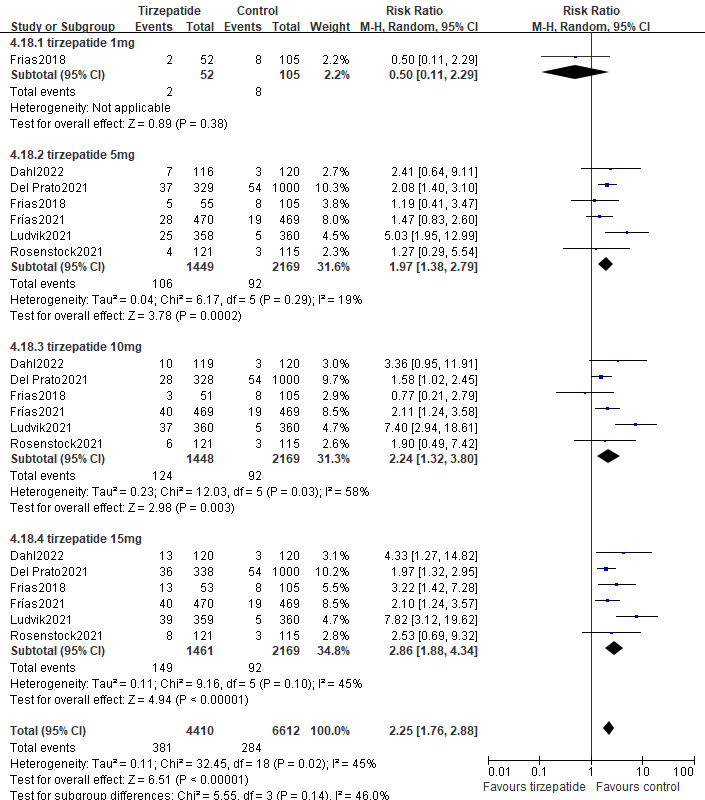


Supplementary Fig. S39 Dose-response of adverse events leading to treatment disontinuation: tirzepatide *vs*. control


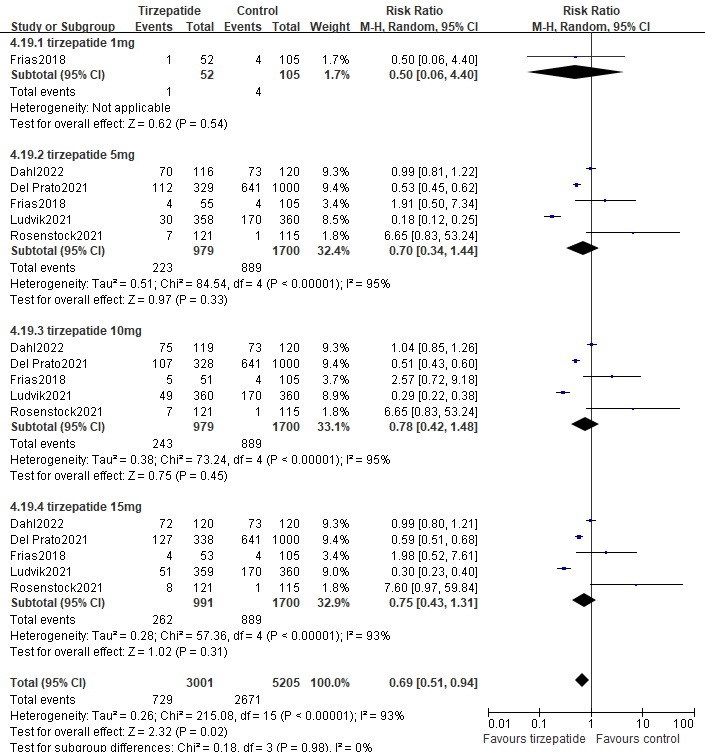


Supplementary Fig. S40 Dose-response of Hypoglycaemia (blood glucose <70 mg/dL): tirzepatide *vs*. control


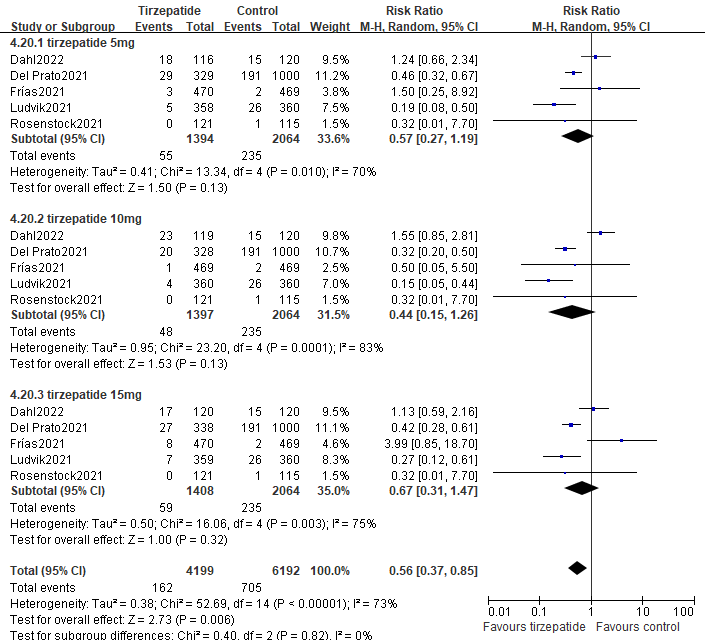


Supplementary Fig. S41Dose-response of Hypoglycaemia (blood glucose <54 mg/dL): tirzepatide *vs.* control


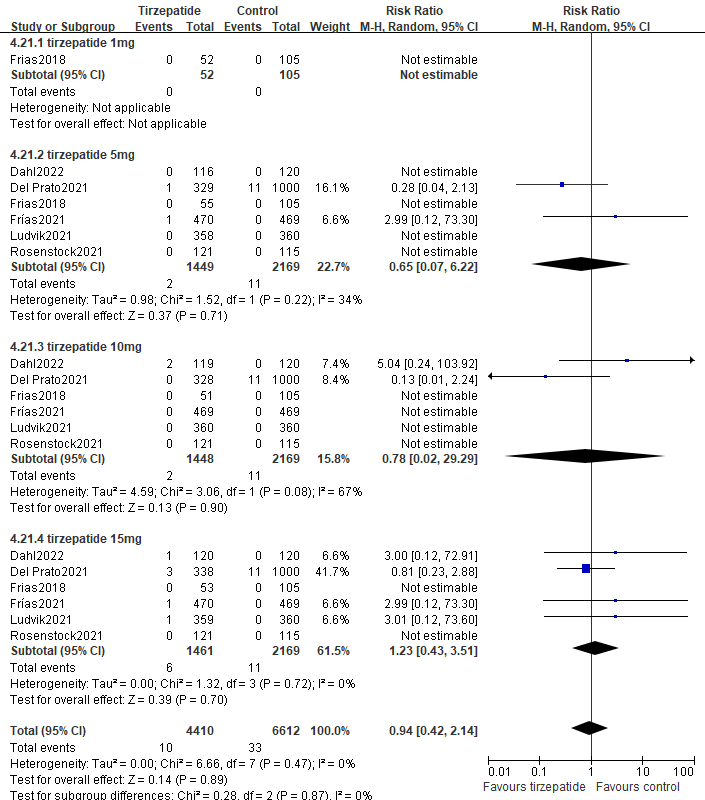


Supplementary Fig. S42 Dose-response of severe hypoglycaemia: tirzepatide *vs*. control


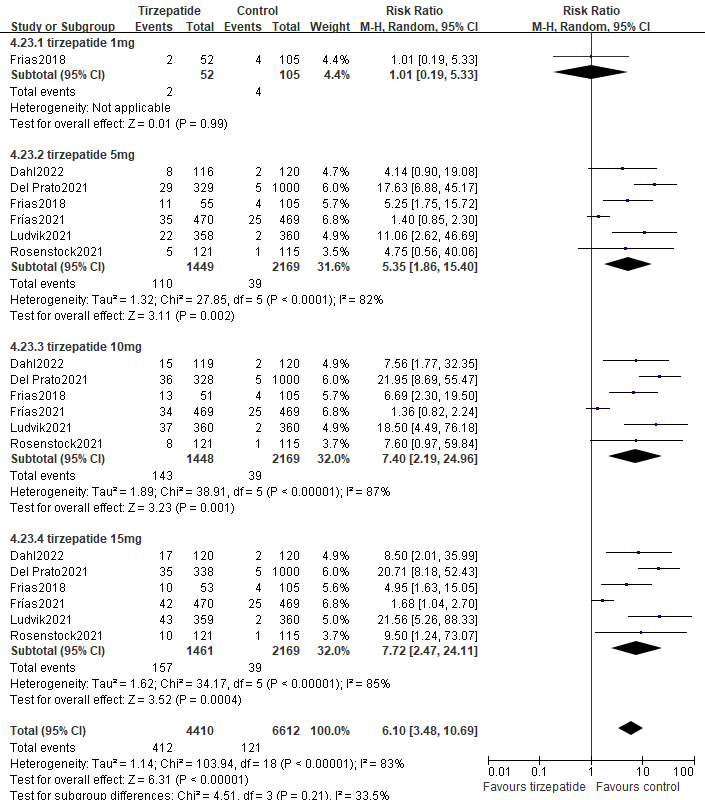


Supplementary Fig. S43 Dose-response of decreased appetite: tirzepatide *vs*. control


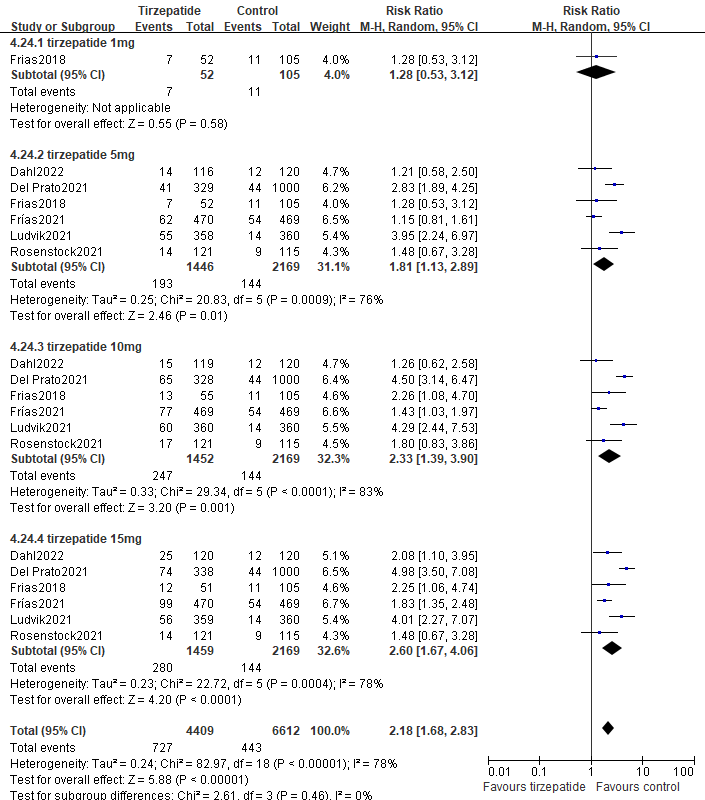


Supplementary Fig. S44 Dose-response of diarrhoea: tirzepatide *vs*. control


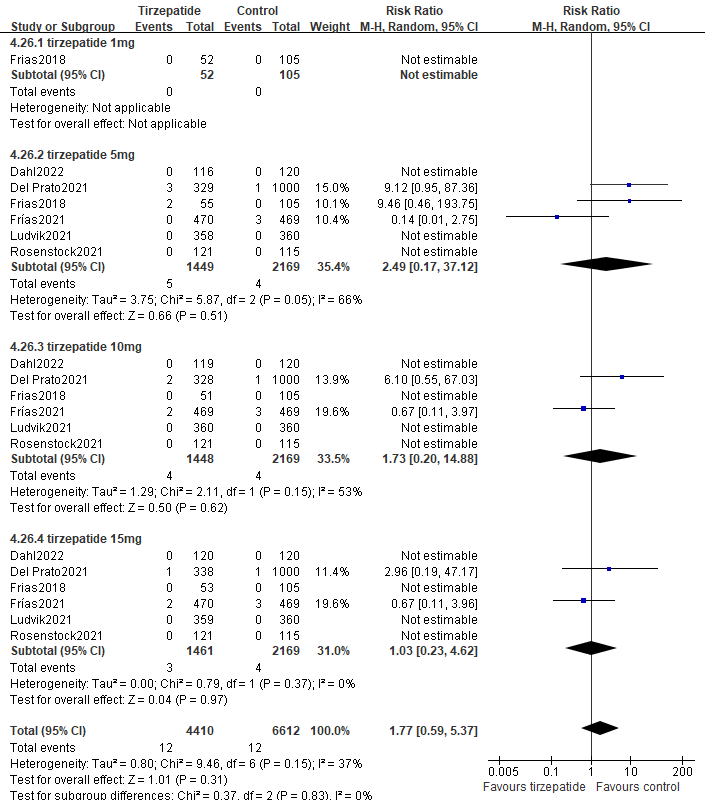


Supplementary Fig. S45 Dose-response pancreatitis: tirzepatide *vs*. control


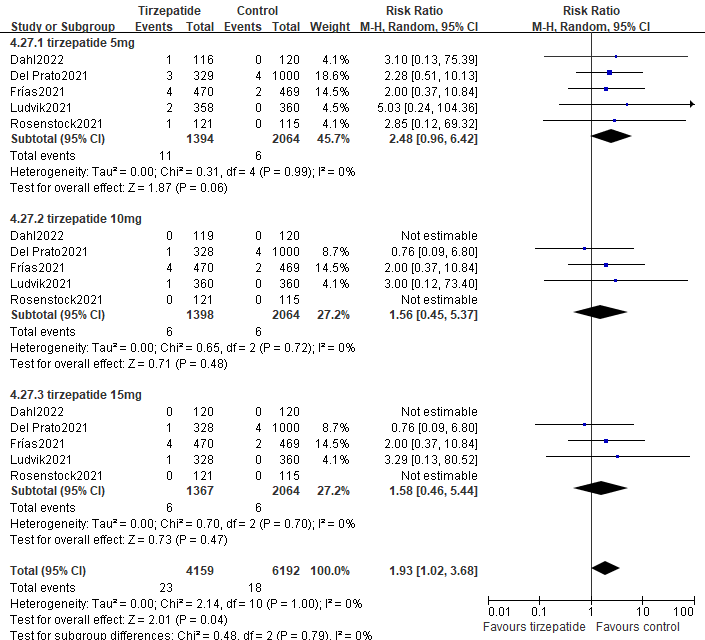


Supplementary Fig. S46 Dose-response cholelithiasis: tirzepatide *vs*. control


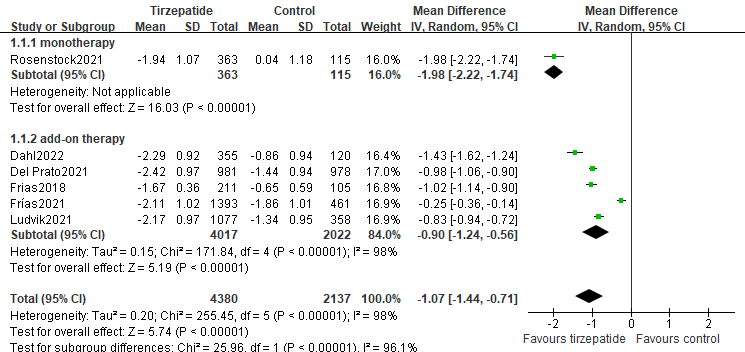


Supplementary Fig. S47 Subgroup analysis of the weighted mean difference in change from baseline in HbA1c based on mono or add-on therapy (%): tirzepatide vs. control.


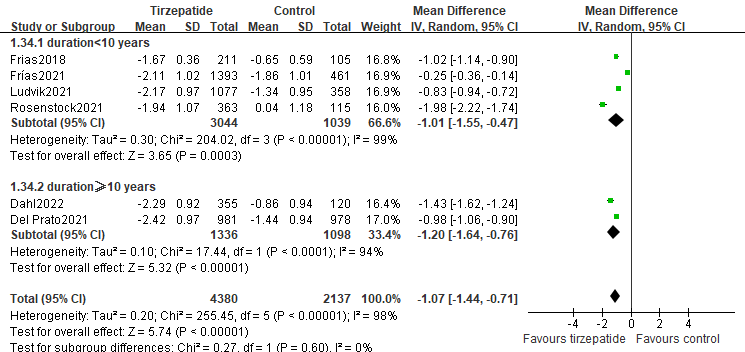


Supplementary Fig. S48 Subgroup analysis of the weighted mean difference in change from baseline in HbA1c based on duration<10years or ≥10 years(%): tirzepatide vs. control.


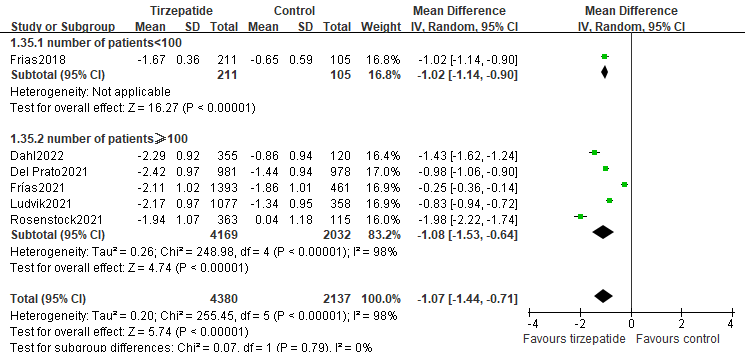


Supplementary Fig. S49 Subgroup analysis of the weighted mean difference in change from baseline in HbA1c based on number of patients <100 or ≥100(%): tirzepatide *vs*. control.

Supplementary Fig. S50 Funnel plot

Supplementary Fig. S51 Begg's funnel plot
